# Redox gradient shapes the abundance and diversity of mercury-methylating microorganisms along the water column of the Black Sea

**DOI:** 10.1128/msystems.00537-23

**Published:** 2023-08-14

**Authors:** Léa Cabrol, Eric Capo, Daan M. van Vliet, F. A. Bastiaan von Meijenfeldt, Stefan Bertilsson, Laura Villanueva, Irene Sánchez-Andrea, Erik Björn, Andrea G. Bravo, Lars-Eric Heimburger Boavida

**Affiliations:** 1 Aix Marseille University, Univ. Toulon, CNRS, IRD, Mediterranean Institute of Oceanography (MIO) UM 110, Marseille, France; 2 Institute of Ecology and Biodiversity (IEB), University of Chile, Santiago, Chile; 3 Department of Marine Biology and Oceanography, Institute of Marine Sciences, CSIC, Barcelona, Spain; 4 Department of Aquatic Sciences and Assessment, Swedish University of Agricultural Sciences, Uppsala, Sweden; 5 Department of Ecology and Environmental Science, Umeå University, Umeå, Sweden; 6 Laboratory of Microbiology, Wageningen University and Research, Wageningen, the Netherlands; 7 Wageningen Food and Biobased Research, Wageningen, the Netherlands; 8 Department of Marine Microbiology and Biogeochemistry, NIOZ Royal Netherlands Institute for Sea Research, Texel, the Netherlands; 9 Faculty of Geosciences, Department of Earth Sciences, Utrecht University, Utrecht, the Netherlands; 10 Department of Chemistry, Umeå University, Umeå, Sweden; University of Hawaii at Manoa, Kaneohe, Hawaii, USA

**Keywords:** mercury methylation, diversity, *hgcAB* gene, metagenomics, redoxcline, niche partitioning, qPCR, MAGs

## Abstract

In the global context of seawater deoxygenation triggered by climate change and anthropogenic activities, changes in redox gradients impacting biogeochemical transformations of pollutants, such as mercury, become more likely. Being the largest anoxic basin worldwide, with high concentrations of the potent neurotoxic methylmercury (MeHg), the Black Sea is an ideal natural laboratory to provide new insights about the link between dissolved oxygen concentration and *hgcAB* gene-carrying (*hgc*^+^) microorganisms involved in the formation of MeHg. We combined geochemical and microbial approaches to assess the effect of vertical redox gradients on abundance, diversity, and metabolic potential of *hgc*^+^ microorganisms in the Black Sea water column. The abundance of *hgcA* genes [congruently estimated by quantitative PCR (qPCR) and metagenomics] correlated with MeHg concentration, both maximal in the upper part of the anoxic water. Besides the predominant *Desulfobacterales*, *hgc*^+^ microorganisms belonged to a unique assemblage of diverse—previously underappreciated—anaerobic fermenters from *Anaerolineales*, *Phycisphaerae* (characteristic of the anoxic and sulfidic zone), *Kiritimatiellales,* and *Bacteroidales* (characteristic of the suboxic zone). The metabolic versatility of *Desulfobacterota* differed from strict sulfate reduction in the anoxic water to reduction of various electron acceptors in the suboxic water. Linking microbial activity and contaminant concentration in environmental studies is rare due to the complexity of biological pathways. In this study, we disentangle the role of oxygen in shaping the distribution of Hg-methylating microorganisms consistently with MeHg concentration, and we highlight their taxonomic and metabolic niche partitioning across redox gradients, improving the prediction of the response of marine communities to the expansion of oxygen-deficient zones.

**IMPORTANCE:**

Methylmercury (MeHg) is a neurotoxin detected at high concentrations in certain marine ecosystems, posing a threat to human health. MeHg production is mainly mediated by *hgcAB* gene-carrying (*hgc*^+^) microorganisms. Oxygen is one of the main factors controlling Hg methylation; however, its effect on the diversity and ecology of *hgc*^+^ microorganisms remains unknown. Under the current context of seawater deoxygenation, mercury cycling is expected to be disturbed. Here, we show the strong effect of oxygen gradients on the distribution of potential Hg methylators. In addition, we show for the first time the significant contribution of a unique assemblage of potential fermenters from *Anaerolineales*, *Phycisphaerae*, and *Kiritimatiellales* to Hg methylation, stratified in different redox niches along the Black Sea gradient. Our results considerably expand the known taxonomic diversity and ecological niches prone to the formation of MeHg and contribute to better apprehend the consequences of oxygen depletion in seawater.

## INTRODUCTION

Decades of anthropogenic emissions and widespread atmospheric dispersal make mercury (Hg) a contaminant of global concern (1). Hg can be transformed into the neurotoxin methylmercury (MeHg) and detected at high concentrations in certain marine ecosystems, where it bioaccumulates and biomagnifies, ultimately causing severe risks for humans (2). The methylation of Hg^II^ to MeHg is mainly mediated by microorganisms and has primarily been described in anoxic environments such as wetlands, sediments, rice paddies, or animal gut (3). Aside from the *sine qua non* presence and activity of microorganisms producing MeHg, Hg methylation is also controlled by Hg bioavailability, organic matter composition, and oxygen concentrations. Relatively high MeHg concentrations and Hg methylation potential have been reported in oxygen-deficient water columns (4, 5). However, there is still limited knowledge about the microbial key players involved in Hg methylation in marine ecosystems and the environmental conditions constraining their activity (6).

Microbial methylation of Hg has been shown to involve and rely on the enzyme coded by the gene pair *hgcA* and *hgcB* (7). Microorganisms that harbor these genes (*hgcAB* gene-carrying [*hgc*^+^] microorganisms), and the methylation capacity they provide, have been identified across diverse microbial lineages and different environments (8 - 10). Most cultivated lineages with experimentally validated Hg methylation capacity have been affiliated to sulfate-reducing, iron-reducing, and/or syntrophic-fermenting bacteria from *Desulfobacterota* and *Firmicutes*, as well as to methanogenic *Archaea* (3, 11, 12). Recent high-throughput sequencing analyses, including the reconstruction of metagenome-assembled genomes (MAGs) from environmental samples, have revealed a broader diversity of putative Hg methylators including previously unknown and largely uncultured *hgc*^+^ microorganisms such as *Planctomycetota*, *Verrucomicrobiota*, *Chloroflexota,* and *Nitrospirota* (13 - 16)*,* some of them being found abundantly in coastal and “dead zone” systems, such as the fully anoxic bottom waters of a stratified fjord (10), a seasonally anoxic fjord (Saanich Inlet) (17), and oxygen-deficient brackish water from the Baltic Sea (18, 19). These studies corroborate that the main methylators differ between ecosystems and along dominant redox gradients.

In the ocean, climate change and human activities lead to current—and further expected—seawater deoxygenation and expansion of oxygen-deficient zones (20). The modification of redox gradients can dramatically affect microbial and biogeochemical cycles, including Hg transformations. Since the Black Sea is the largest and deepest permanently euxinic (anoxic and sulfidic conditions) stratified basin worldwide, with stable redox gradients extending over several tens of meters (21, 22), it is a promising target to study the partitioning of Hg-methylating microorganisms over contrasted redox niches. A stable permanent pycnocline prevents mixing and exchange between the upper oxygenated layers and anoxic deep waters, separated by a suboxic water layer extending from 75 to 120 m (down to 240 m at certain coastal stations) (23). In the Black Sea water column, high concentrations of MeHg concentrations have been reported in the suboxic and anoxic water layers (24, 25). The high horizontal (isopycnal) homogeneity of the Black Sea has been demonstrated, in terms of microbial community composition (26), hydrography (27), and geochemistry, including Hg chemistry (25). However, the microorganisms involved in Hg methylation in the Black Sea water column have not been identified yet, neither the effect of the vertical stratification on their composition. As reported for other euxinic systems (e.g., Baltic Sea, Cariaco Basin, Saanich Inlet), it is plausible that the vertical gradient of oxygen and HS^−^ concentrations in the Black Sea shapes the distribution of microbial communities, including those involved in MeHg formation, as well as their associated metabolic and biogeochemical functions (22, 28 - 30).

In this study, we aimed to determine the impact of the vertical redox gradient on the microorganisms potentially responsible for Hg methylation in the water column of the Black Sea. We combined geochemical and molecular data obtained from two sampling campaigns conducted in 2013 that had resulted in the previous characterization of (i) high-resolution Hg and MeHg concentrations throughout full water column transects (25) and (ii) specific (sulfur- and organic matter-linked) microbial metabolisms through genome-centric metagenomics (29 - 31). We used several molecular analyses including 16S rRNA amplicon sequencing, metagenomics, and clade-specific quantitative PCR of the *hgcA* gene, at different depths, to determine whether the highest concentrations of MeHg observed in the anoxic water layers of the Black Sea were explained by the presence, abundance, and metabolic traits of *hgc*^+^ microorganisms. The present study is novel in revealing the oxygen-dependent niche partitioning of diverse microorganisms potentially capable of Hg methylation in the Black Sea, consistently with measured MeHg concentrations.

## MATERIALS AND METHODS

### Sampling campaigns

Two complementary field cruises were conducted almost simultaneously in June–July 2013 in the Black Sea with the vessel R/V Pelagia to obtain (i) Hg chemistry data, *hgcA* quantitative PCR (qPCR), and 16S rRNA gene amplicon sequencing data (MEDBlack cruise) and (ii) planktonic metagenomes (Phoxy cruise). Following the definitions in Stewart et al. (32) and the vertical discretization model of Rosati et al. (25), the water column was decomposed into three redox layers: the oxic layer (OL, from 0 to 75 m depth, characterized by O_2_ concentrations between 30 and 300 µM and undetectable HS^−^ concentrations), the suboxic layer (SOL, from 75 to 120 m depth, characterized by dissolved O_2_ concentrations <20 µM and HS^−^ concentrations <5 µM), and the anoxic layer (AOL, below 120 m depth, characterized by undetectable O_2_ concentrations and HS^−^ concentrations between 15 and 400 µM).

**Fig 1:**
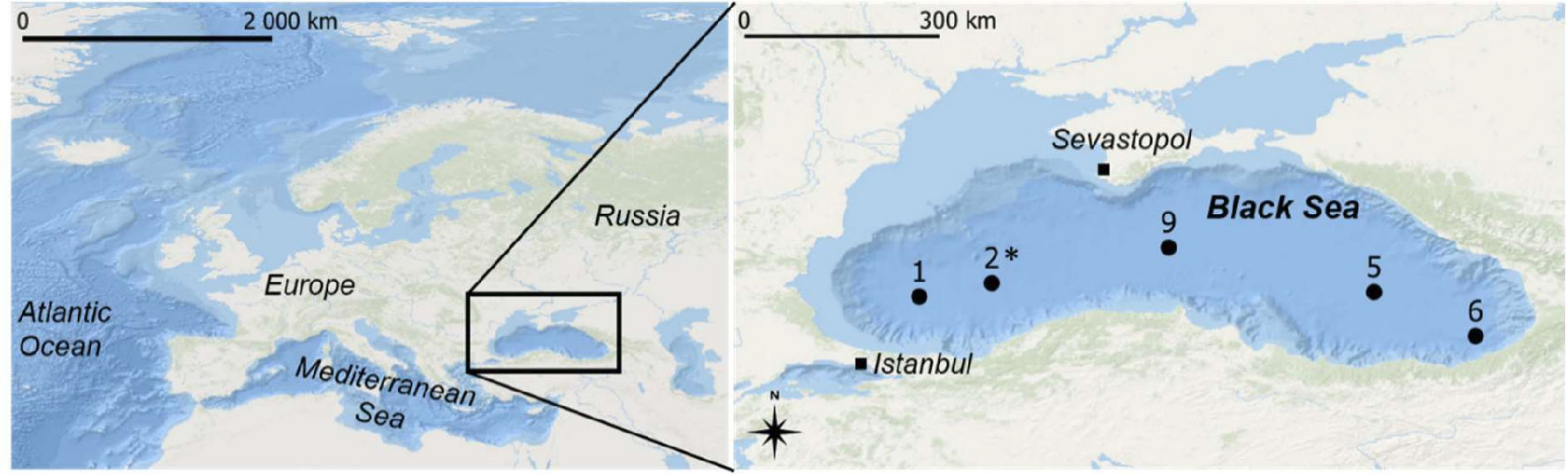
Locations of stations sampled in the Black Sea during the MEDBlack cruise and analyzed in this study (black dots). The star indicates station 2 which was also sampled during the Phoxy cruise for metagenomics analysis. The exact coordinates are provided in Data Sheet S1A.

The MEDBlack cruise was conducted from 13 to 25 July 2013 and occupied 12 stations along a west-to-east transect. In the present work, we include five stations (1, 2, 5, 6, and 9, according to previous nomenclature [25]; Fig. 1). For microbiology, water samples were collected from three water depths: OL (30–50 m), SOL (90–110 m, except coastal station 6 sampled at 180 m depth), and AOL (140–150 m, except coastal station 6 sampled at 250 m depth) (exact sampling depths are provided in Data Sheet S1A). Samples were filtered with *in situ* Stand Alone Pump System (Challenger Oceanic, from NOC, Southampton) equipped with two 293 mm diameter filters: a Petex nylon mesh pre-filter (51 µm; 150 µm for station 1) and a polycarbonate filter (1 µm) further stored at −20°C.

The Phoxy cruise 64PE371 was conducted on 9 and 10 June 2013 in the western gyre of the Black Sea. Suspended particulate matter was collected from 15 depths across the oxygen gradient in the water column (from 50 to 2,000 m depth) at sampling station 2 (42.89 N, 30.67 E, 2,107 m depth [Fig. 1], at 72 km from the MEDBlack station 2 and visited 35 days earlier) with McLane WTS-LV *in situ* pumps (McLane Laboratories Inc., Falmouth, MA, USA) on pre-combusted glass fiber filters with 142 mm diameter and 0.7 µm nominal pore size, further stored at −80°C. One sample from the Phoxy cruise belonged to the OL (50 m depth), eight samples to the SOL (70–110 m depth), and six samples to the AOL (130–2000,m depth).

### Physicochemical measurements

For the Phoxy cruise, chemical parameters included the dissolved concentrations of O_2_, HS^−^, NH_4_^+^, NO_2_^−^, and NO_3_^−^ (Data Sheet S1B). Dissolved O_2_ concentration was measured by a conductivity-temperature-depth (CTD) probe equipped with a Seabird SBE 43 electrochemical O_2_ sensor which was calibrated against on-deck Winkler titrations, with a detection limit of 2 µM. Nutrients concentrations were measured on a QuAAtro autoanalyzer with a detection limit of 0.26, 0.031, 0.011, 0.007, and 0.008 µM for HS^−^, NH_4_^+^, NO_3_^−^, NO_2_^−^, and PO_4_^3−^, respectively (33). A summary of available and new data for both campaigns is provided in Table 1.

**TABLE 1:** Description of samples included in this study and summary of the main measured geochemical parameters, available from Rosati et al. (25) (for MEDBlack samples) and from Sollai et al. (33) (for Phoxy cruise)^*a*^

Sample name	Cruise campaign	Sampling depth (m)	Concentrations of dissolved elements						Method
			O_2_ (µM)	HS^−^ (µM)	NH_4_^+^ (µM)	PO_4_^3−^ (µM)	tHg (pM)	MeHg (pM)	
F1-OL	MEDBlack	55.5	142.4	<LoD	<LoD	0.7	1.4	0.2	MB, qPCR
F1-SOL	MEDBlack	85.7	3.4	<LoD	0.1	0.9	1.4	0.1	MB, qPCR
F1-AOL	MEDBlack	145.3	<LoD	19.3	13.5	5.0	3.1	0.8	MB, qPCR
F2-OL	MEDBlack	24.2	423.0	<LoD	0.2	<LoD	3.3	0.1	MB, qPCR
F2-SOL	MEDBlack	99.5	1.8	<LoD	3.9	6.2	1.6	0.2	MB, qPCR
F2-AOL	MEDBlack	144.8	<LoD	22.6	15.2	5.0	5.3	0.8	MB, qPCR
F9-OL	MEDBlack	45.8	213.1	<LoD	0.1	0.8	2.2	0.1	MB, qPCR
F9-SOL	MEDBlack	100.4	1.8	<LoD	2.0	6.6	2.2	0.2	MB, qPCR
F9-AOL	MEDBlack	149.6	<LoD	17.7	15.8	4.8	3.6	1.1	MB, qPCR
F5-OL	MEDBlack	40.5	373.8	<LoD	0.1	<LoD	2.8	0.1	MB, qPCR
F5-SOL	MEDBlack	110.4	1.8	<LoD	3.6	7.0	3.2	0.4	MB, qPCR
F5-AOL	MEDBlack	149.4	<LoD	15.3	13.8	4.8	3.3	0.8	MB, qPCR
F6-OL	MEDBlack	40.3	377.0	<LoD	<LoD	<LoD	3.0	0.1	MB, qPCR
F6-SOL	MEDBlack	175.0	10.7	<LoD	0.2	3.1	2.9	0.4	MB, qPCR
F6-AOL	MEDBlack	250.3	<LoD	36.5	19.2	4.9	3.3	0.8	MB, qPCR
F2-50	Phoxy	50	121.2	<LoD	0.1	0.7	Nd	Nd	MG
F2-70	Phoxy	70	2.2	<LoD	0.1	1.1	Nd	Nd	MG
F2-80	Phoxy	80	<LoD	<LoD	0.1	1.1	Nd	Nd	MG
F2-85	Phoxy	85	<LoD	<LoD	0.1	0.8	Nd	Nd	MG
F2-90	Phoxy	90	<LoD	<LoD	0.4	2.1	Nd	Nd	MG
F2-95	Phoxy	95	<LoD	<LoD	1.1	4.7	Nd	Nd	MG
F2-100	Phoxy	100	<LoD	<LoD	5.7	7.2	Nd	Nd	MG
F2-105	Phoxy	105	<LoD	0.9	7.2	7.9	Nd	Nd	MG
F2-110	Phoxy	110	<LoD	4.6	8.8	6.7	Nd	Nd	MG
F2-130	Phoxy	130	<LoD	14.7	13.9	5.5	Nd	Nd	MG
F2-170	Phoxy	170	<LoD	31.6	20.1	4.9	Nd	Nd	MG
F2-250	Phoxy	250	<LoD	84.7	32.6	5.2	Nd	Nd	MG
F2-500	Phoxy	500	<LoD	206.3	59.8	6.8	Nd	Nd	MG
F2-1000	Phoxy	1,000	<LoD	353.8	90.8	7.9	Nd	Nd	MG
F2-2000	Phoxy	2,000	<LoD	397	100.2	8.4	Nd	Nd	MG

^*a*^ Detailed data sets including additional measured parameters are provided in Data Sheet S1A and B. Nd, non-determined; LoD, limit of detection. The “method” column stands for the molecular methods applied in the current study: MB for metabarcoding, qPCR for quantitative PCR, MG for metagenomics.

For the MEDBlack cruise, available measured physicochemical parameters (Data Sheet S1A) include pressure, temperature, conductivity, fluorescence, salinity, density, and the dissolved concentrations of O_2_, HS^−^, NH_4_^+^, NO_2_^−^, NO_3_^−^, PO_4_^3−^, Si, Fe, DOC, total Hg, and MeHg (sum of mono- and di-MeHg) according to previous protocols (25). Specific analysis of Hg and MeHg is detailed in Supplementaal Information 1.1. The detection limit was 0.025 and 0.001 pM for HgD and MeHgD, respectively. Nutrients, HS^−^, and dissolved O_2_ concentrations were measured similarly to the Phoxy cruise with the same detection limits. Despite coming from two different cruises, the variability between nutrients, HS^−^, and dissolved O_2_ concentrations measured in both cruises at similar depths is low (4–14% variability on average).

### DNA extractions

From the MEDBlack cruise, DNA was extracted from sections of the 15 filters (i.e., five stations, three depths) with the FastDNA kit and FastPrep homogenizer (MP Biomedicals, Santa Ana, CA, USA) according to the manufacturer instructions. The filter fraction represented 2.5% of the whole filter area, corresponding to approximately 5–13 L of filtered seawater depending on the sampling points (8 L on average). From the Phoxy cruise, DNA was extracted from sections of 15 glass fiber filters (1/8 filter from 50 to 130 m depth and 1/4 from 170 to 2,000 m depth) with the RNA PowerSoil Total Isolation Kit plus the DNA elution accessory (Mo Bio Laboratories, Carlsbad, CA, USA) as previously described (31).

### Amplicon sequencing and qPCR estimates of 16S rRNA genes

For the MEDBlack cruise DNA samples, the hypervariable V4-V5 region of the 16S rRNA gene from *Bacteria* and *Archaea* was amplified with high-fidelity Phusion Hot Start II DNA polymerase (Thermo Scientific, Waltham, MA, USA) using universal primers 515F and 928R (34) and a two-step PCR protocol (35), as detailed in Supplemental Information 1.2. and Table S1. The correct amplicon size and the absence of non-specific bands were checked by agarose gel electrophoresis. Amplicons were sequenced using a 2 × 250 bp paired-end MiSeq system (Illumina, USA) at the Genotoul platform (Toulouse, France). The raw sequences have been deposited at NCBI GenBank, SRA database, under the BioProject accession number PRJNA895066.

Raw sequences were analyzed on the Galaxy bioinformatics platform through the FROGS pipeline, version 3.2.3, as detailed in the Supplemental Information. Especially, operational taxonomic units (OTUs) were defined by sequence clustering, using the high-resolution SWARM algorithm v3.2.3 (36). After filtering at 0.005% of abundance, OTUs were taxonomically annotated with the SILVA 16S database (version 138.1).

Bacterial and archaeal abundances were quantified in the MEDBlack cruise DNA samples by quantitative PCR with Takyon No Rox SYBR 2X Master Mix (Eurogentec, Seraing, Belgium). Protocol details are provided in Table S1.

### Clade-specific *hgcA* gene qPCR estimates and cloning sequencing of *hgcA* sequences

In the MEDBlack cruise DNA samples, the *hgcA* genes of each of the three dominant Hg-methylating clades (*Desulfobacterota*, *Firmicutes*, and *Archaea*) were quantified by qPCR, using clade-specific degenerated qPCR primers (37) and Takyon No Rox SYBR 2X Master Mix (Eurogentec, Seraing, Belgium). The qPCR conditions have been optimized as detailed in the Supplemental Information. All qPCR details and primer sequences are provided in Supplemental Information 1.3. and Table S1.

For *Archaea-hgcA*, due to the low amplification efficiency and the ambiguity in the melting curves and agarose gel electrophoresis, the correct affiliation of the *hgcA* amplicons was verified by cloning and sequencing the amplified PCR product, as detailed in Supplemental Information 1.4. Obtained DNA sequences were translated into amino acid sequences and compared with *hgcA* sequences from the Hg-MATE database (38) and from the 15 metagenomes obtained in the Phoxy cruise (see following section). The sequence analysis (Supplemental Information 1.4 and Fig. S2) showed that *hgcA* sequences obtained with this primer set clustered with *Euryarchaeota* and *Chloroflexota* sequences. Thus, from thereon, *hgcA* qPCR estimates from *Archaea* will be referred to *hgcA* qPCR estimates from *Archaea-Chloroflexota*.

### Metagenomic estimates of *hgcA* genes

The 15 DNA extracts from the Phoxy cruise were used to prepare TruSeq nano libraries, sequenced with Illumina MiSeq (five samples multiplexed per lane) at Utrecht Sequencing Facility, generating 4.5 × 10^7^ paired-end reads (2 × 250 bp), which were further processed as previously reported (31) and summarized in Supplemental Information 1.5.

*hgc* homologs were identified and annotated in the amino acid FASTA file (generated from protein-coding genes detected in the metagenomes coassembly), using the HMM profiles of *hgcA* and *hgcB* genes derived from the Hg-MATE database v1.01142021 (38) and the function *hmmsearch* from HMMER 3.2.1 (39). We considered genes with *E*-values <10^−3^ as significant hits. To further confirm putative *hgcA* and *hgcB* genes (or hgc-like genes) within the HMM search hits, we used the high stringency cutoff defined by Capo et al. (8) by looking for unique conserved motifs from *hgcA* gene (NVWCA(A/G/S)GK) and performed a manual inspection of the presence of *hgcA* genes. Coverage values of *hgcA* genes were calculated as the number of reads mapped to the gene divided by its length (base pairs, bp) and were further normalized by dividing them by the coverage values of the marker gene *rpoB*. The *rpoB* genes were detected using the function *hmmsearch* from HMMER 3.2.1 (39) and HMM profile TIGR02013.hmm for bacterial *rpoB* genes and applying the trusted cutoff provided in HMM files.

The obtained *hgcA* homologs, translated in amino acid sequences, were taxonomically affiliated through a phylogenetic analysis, by placing them onto the HgcA reference tree using a *pplacer* approach (40). The construction of the reference tree was based on the reference package “hgcA” from Hg-MATE database v1, according to the protocol in the README.txt of the Hg-MATE database (38) and in Capo et al. (8), as detailed in Supplemental Information 1.5. As a complement to this community-level analysis, metagenome-assembled genomes generated from the same previously published metagenomes (30, 31) were screened for *hgc* genes. The *hgc* genes found in MAGs were taxonomically identified using the MAG phylogeny and taxonomy (Data Sheet S1D).

### Data analysis

The qPCR estimates were statistically analyzed by two-way analysis of variance (ANOVA) with the *aov* function (R software). The selected environmental parameters for correlation analysis were the concentrations of dissolved O_2_, HS^−^, and MeHg. Spearman correlation plots were obtained using the functions *rcorr* and *corrplot* from the R packages Hmisc and *corrplot*, respectively. Shannon diversity indices were calculated with the *estimate_richness* function in the *vegan* R package. Principal coordinates analysis (PCoA) was performed applying the function *wcmdscale* to Bray-Curtis dissimilarity matrixes built with the function *vegdist* from the 16S rRNA gene-based OTU abundance table and the *hgcA* gene abundance (normalized coverage values) in the R package *vegan*. The discriminant *hgcA* genes explaining the most the clustering of *hgc*^+^ community according to depth zones were identified by linear discriminant analysis (*run_lefse* function, *microbiomeMarker* package) with cumulative sum scaling normalization and 100 bootstraps.

## RESULTS

### Mercury and microbial community stratification along water depth and oxygen gradient

Hg and MeHg profiles revealed a strong depth stratification, homogeneous across the whole basin (Fig. S1). In stations 1, 2, and 5, the MeHg concentration was always maximal at the same depth (130 m) reaching 0.83–1.13 pM, which represents 32%–48% of tHg at this depth. The MeHg concentration then decreased down to 250 m depth and remained relatively stable in deeper waters (0.50–0.65 pM on average below 250 m depth, representing 19%–26% of tHg).

**Fig 2:**
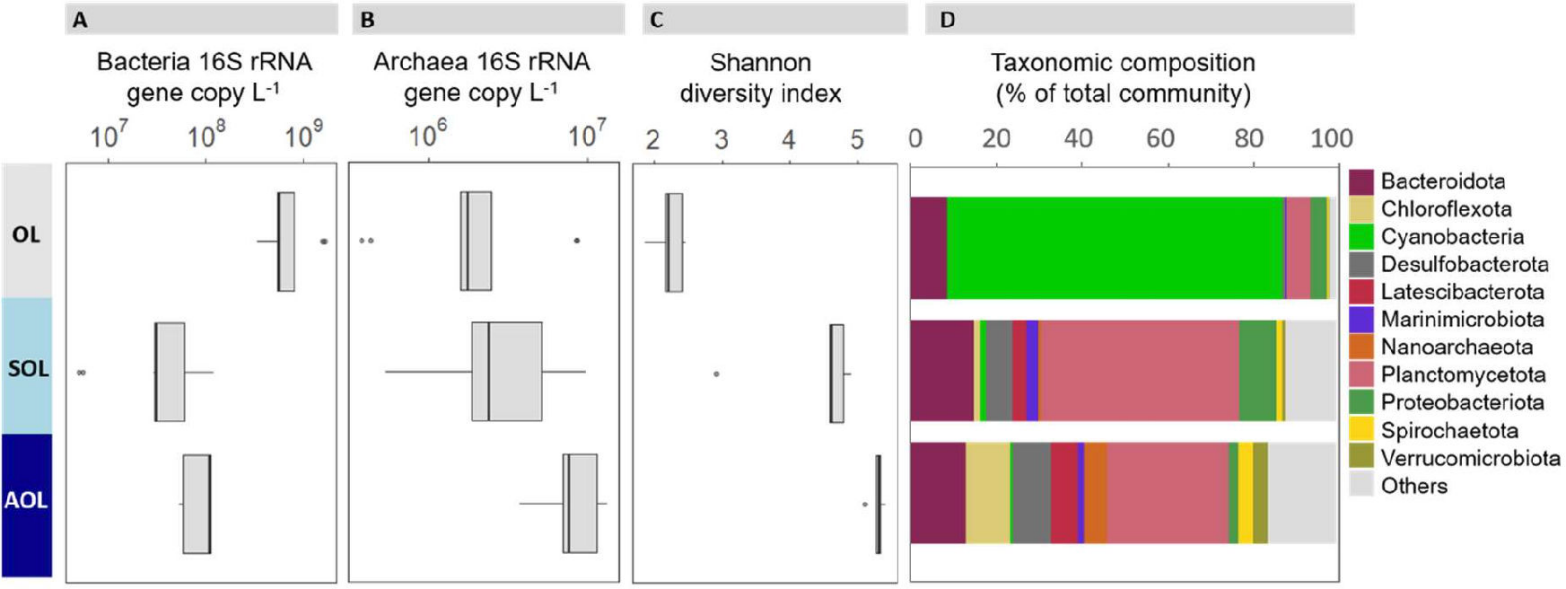
Stratification of microbial profiles in the Black Sea water column. Data from the five sampling stations have been averaged for oxic (OL, 25–50 m), suboxic (SOL, 85–110 m, except station 6 at 175 m), and anoxic (AOL, 145–250 m) layers. (A) qPCR quantification of bacterial 16S rRNA gene, per filtered volume of seawater. (B) qPCR quantification of archaeal 16S rRNA gene, per filtered volume of seawater. (C) Diversity of total microbial community based on Shannon index, computed from 16S rRNA gene amplicon sequences. (D) Taxonomic composition of microbial communities at the phylum level, representing the 11 most abundant phyla. In panels A to C, the bold line of each boxplot shows the median, while upper and lower limits of the boxes represent the first and third quartiles, respectively. The whiskers are maximum and minimum values and the dots show the outliers. For panel D, for each phylum, the averages and standard deviations calculated per water layer are provided in Data Sheet S1E. The taxonomic composition of the five individual sampling stations across the Black Sea basin is shown in Fig. S3 (at the phylum level) and the detailed OTU composition is provided in Data Sheet S1C.

Microbial community structure was homogeneous horizontally across the Black Sea basin, but vertically stratified across water depth and oxygen concentrations. Considering the MEDBlack samples, bacterial abundances based on qPCR of the bacterial 16S rRNA gene did not significantly differ along the west-to-east transect (two-way ANOVA, *P* = 0.4) but were significantly impacted by sampling depth (two-way ANOVA, *P* < 0.001) (Fig. 2A; Data Sheet S1A). The highest bacterial abundances (7.8 10^8^ 16S copies L^−1^ on average) were observed in the OL (30–50 m depth), being one order of magnitude higher than in the AOL (140–250 m depth). Archaeal abundances measured by qPCR also varied with depth (*P* = 0.001), but showed a reverse stratification pattern from bacteria (Fig. 2B) as detailed in Supplemental Information 2.1.

**Fig 3:**
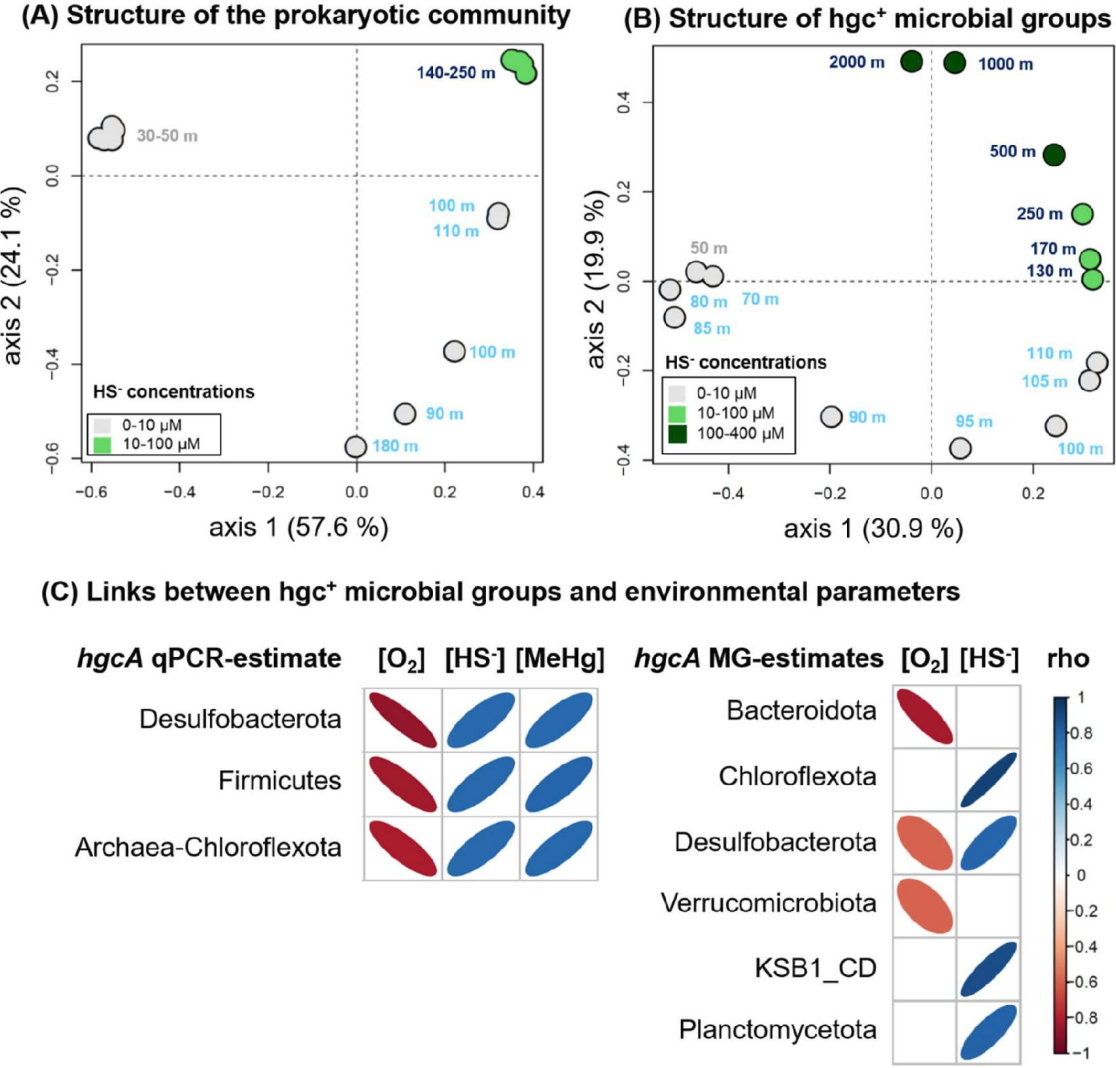
Depth stratification of prokaryotic and *hgc*^+^ communities in relation with environmental gradients and Hg-related variables. (**A and B**) PCoA plots based on Bray-Curtis dissimilarity showing the structure of the overall prokaryotic community (A; MEDBlack cruise samples) and of the *hgc*^+^ microbial groups (B; Phoxy cruise samples). The dot color denotes the HS^−^ concentrations measured in the same samples. The color of the sample name label corresponds to the three water-depth zones: the oxic (OL, gray), suboxic (SOL, light blue), and anoxic (AOL, dark blue) water layers. (C) Correlation plots based on Spearman correlation coefficients between environmental parameters (O_2_, HS^−^, and MeHg concentrations) and qPCR and metagenomic (MG) estimates of *hgcA* genes from different microbial groups. Only significant relationships (*P* < 0.05) are displayed. No MeHg measurements were done during the Phoxy cruise (metagenome samples).

The taxonomic composition (Fig. S3) and the structure (Fig. 3A) of the prokaryotic community was similar across the west-east transect, as confirmed by the non-significant effect of sampling station on the community structure permutational multivariate analysis of variance (PERMANOVA *P* > 0.1) and Shannon diversity (ANOVA, *P* = 0.16). For all stations, OL communities were dominated by *Cyanobacteria* representing 70% of prokaryotes (Fig. 2D; Fig. S3; Data Sheet S1C and E). SOL and AOL were significantly more diverse (ANOVA, *P* < 0.001, Fig. 2C), dominated by *Planctomycetota* (29%–47% of the prokaryotes), *Bacteroidota* (13%–15%), and *Desulfobacterota* (7%–9%) (Supplemental Information 2.1).

### Clade-specific *hgcA* genes measured by qPCR are more abundant in the anoxic layer

**Fig 4:**
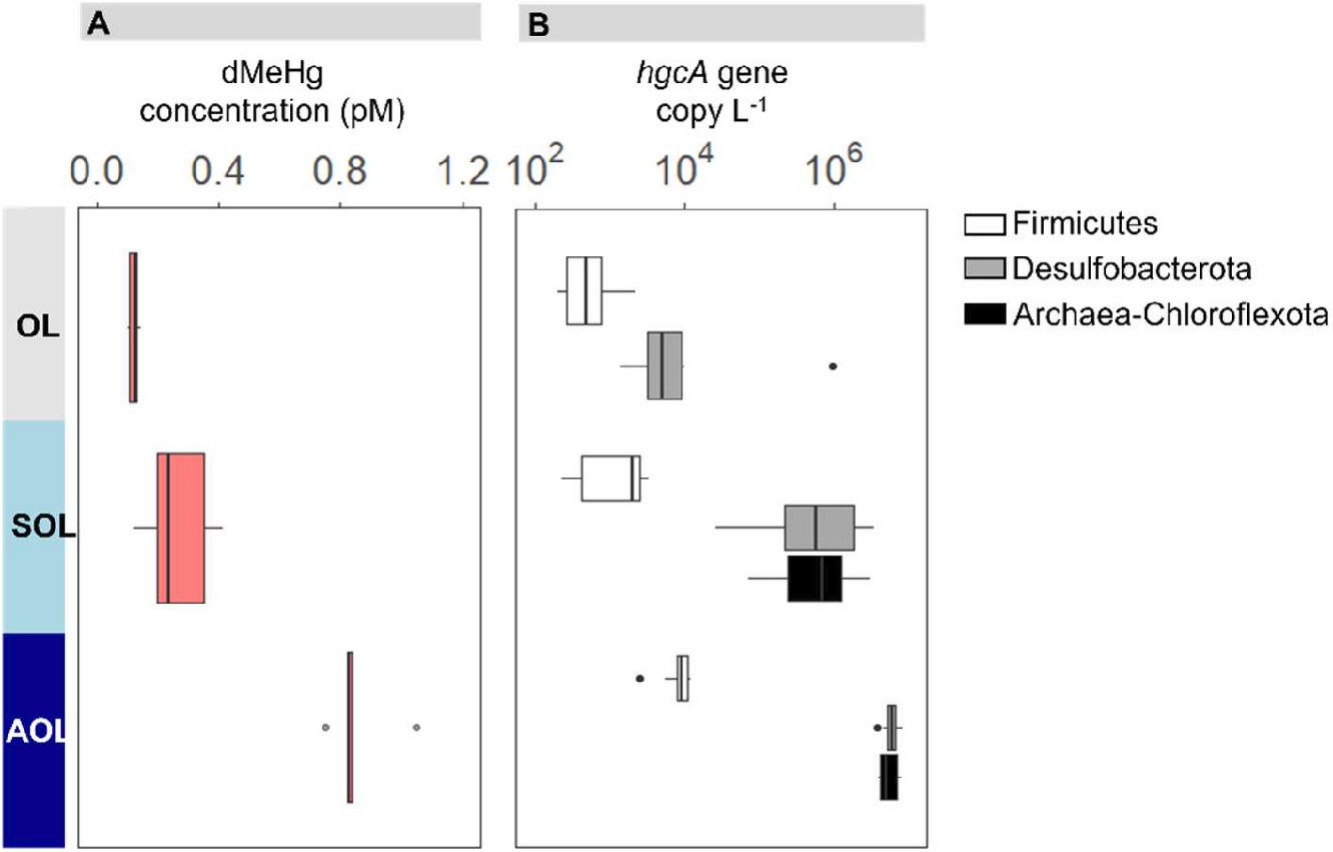
Vertical stratification of MeHg concentrations and *hgcA* gene abundances in the Black Sea water column. Data from the five sampling stations have been averaged for oxic (OL, 25–50 m), suboxic (SOL, 85–110 m, except station 6 at 175 m), and anoxic (AOL, 145–250 m) layers. (A) Dissolved concentration of MeHg. (B) qPCR quantification of *hgcA* gene, indicator of Hg methylation ability, for *Firmicutes*, *Desulfobacterota,* and *Archaea-Chloroflexota*, in copies per seawater filtered volume. The bold line of each boxplot shows the median, while upper and lower limits of the boxes represent the first and third quartiles, respectively. The whiskers are maximum and minimum values and the dots show the outliers. The values for the five individual sampling stations are provided in Data Sheet S1A, as well as the relative *hgcA* counts (normalized by total prokaryotic abundance).

Abundance of *hgcA* genes was quantified by qPCR for each of the well-known Hg-methylating clades *Desulfobacterota*, *Firmicutes*, and *Archaea-Chloroflexota* (37) (see Supplemental Information 1.4 for the clade definition). Analogous to 16S rRNA gene counts, absolute *hgcA* counts from *Desulfobacterota*, *Firmicutes,* and *Archaea-Chloroflexota* were similar along the west-to-east transect of the Black Sea (two-way ANOVA, *P* > 0.90), but strongly stratified over depth (two-way ANOVA, *P* < 0.0001). Tracking the MeHg concentration profiles (Fig. 4A; Fig. S1), the highest *hgcA* counts were found in the AOL for all three clades, reaching on average 9.0 ± 3.0 10^3^, 6.1 ± 1.4 10^6^, and 5.6 ± 1.5 10^6^ copies L^−1^ of seawater for *Firmicutes*, *Desulfobacterota,* and *Archaea-Chloroflexota,* respectively (Fig. 4B; Data Sheet S1A). The *hgcA* counts were between 12 and 31 times lower in the OL. In the SOL and AOL, *hgcA* counts from *Desulfobacterota* and *Archaea-Chloroflexota* were similar, exceeding that of *Firmicutes* by one to three orders of magnitude.

The higher abundance of *hgcA* genes in the AOL was not only observed for absolute *hgcA* counts, but also for relative *hgcA* counts (normalized by summed bacterial and archaeal 16S gene counts, Data Sheet S1A), which contrasts with the pattern observed for total bacteria and indicates a clear enrichment of potential Hg methylators in oxygen-deficient deeper waters. Relative *hgcA* counts from *Desulfobacterota* represented 4.8%–9.8% of the prokaryotes in the AOL, 0.5%–2.9% in the SOL, and <0.2% in the OL. *hgcA* counts from *Archaea-Chloroflexota* followed a similar depth pattern, representing 4.3%–6.9% of the prokaryotes in the AOL and 0.8%–2% in the SOL, while not detected in the OL. Finally, *Firmicutes-hgcA* genes accounted for <0.02% of the prokaryotes in all layers. The specificity of the *Archaea-Chloroflexota hgcA* primers is further evaluated and discussed in the Supplemental Information.

### *hgc* genes in the water column are affiliated to diverse taxa with specific niches

**Fig 5:**
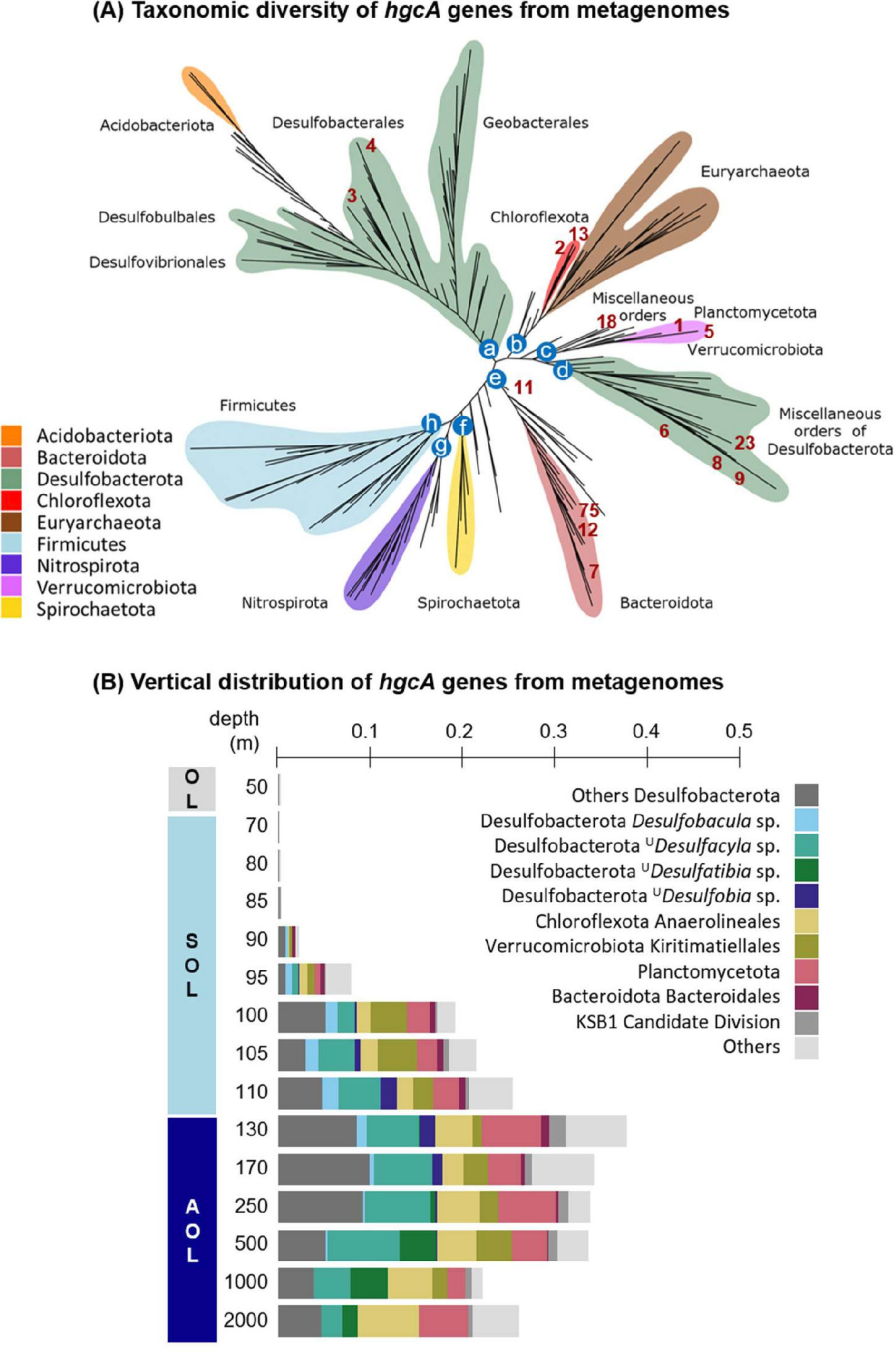
A diverse microbial community potentially able to methylate Hg in the Black Sea water column. (**A**) Unrooted *hgcA* phylogenetic tree showing the diversity of *hgc*^+^ microorganisms from the Black Sea water column compared to the reference sequences from the Hg-MATE database. The 15 binned *hgcA* genes found in MAGS are denoted in the figure by their ID number in red (see “gene_id” in Data Sheet S1D). The other 77 unbinned *hgcA* genes are listed in Data Sheet S1D; after examination of their placement on the phylogenetic tree, they were assigned to a taxonomic cluster denoted from “a” to “h” (indicated in the column “Corresponding cluster in *hcgA* tree” of Data Sheet S1D). For clarity purpose, only the cluster letter is shown on the tree (blue circles at the basis of each group) instead of the 77 sequences. The reference phylogenetic tree was provided by the Hg-MATE database using *hgcA* sequences from (i) pure culture/environmental microbial isolates (204 sequences), (ii) single-cell genome sequences (29 sequences), and (iii) metagenome-assembled genomes (787 sequences), as detailed in Materials and Methods and Supplemental Information 1.5. (**B**) Vertical distribution of MG-estimated coverage values of *hgcA* genes normalized by *rpoB* coverage values along the water column depth in the Black Sea. The corresponding compartmentation into oxic (OL), suboxic (SOL), and anoxic (AOL) layers is shown on the left axis. Color code corresponds to taxonomic affiliation.

A total of 91 *hgcA* genes were found in the Black Sea metagenome co-assembly, including 14 genes found next to *hgcB* genes on the same contig (Data Sheet S1D). Among them, 15 *hgcA* genes were found in MAGs affiliated to various orders of *Desulfobacterota* (*Desulfobacterales*, *Desulfobulbales*, unclassified), *Chloroflexota* (*Anaerolineales*), *Bacteroidota* (*Bacteroidales*), *Planctomycetota* (*Phycisphaerales*), *Verrumicrobiota* (*Kiritimatiellales*), and KSB1 candidate division (unclassified) (Fig. 5A). Among *Desulfobacterota*, the *hgc*^+^ MAGs were identified as uncultured species of ^U^*Desulfacyla*, ^U^*Desufatibia,* ^U^*Desulfobia,* and *Desulfobacula*. From the alignment to the Hg-MATE database, the remaining 76 unbinned *hgcA* genes were affiliated predominantly to *Desulfobacterota* (32), *Planctomycetota* (14, among which 4 *Phycisphaerae*), *Verrucomicrobiota* (9 *Kiritimatiellales*), *Chloroflexota* (8 *Anaerolineales*), and various microbial lineages (Data Sheet S1D).

The metagenome-estimated (MG-estimated) abundance and diversity of *hgcA* genes were both strongly stratified along the Black Sea water column (Fig. 5B; Data Sheet S1B). This was in line with the qPCR results for clade-specific *hgcA* genes, although samples for metagenomic analysis were collected with a higher vertical resolution (15 depths). *hgcA* genes were barely detected in the OL and at the top of the SOL (<0.1% of the reads above 70 m depth). The abundance of *hgcA* genes (i.e., normalized coverage values) gradually increased over depth, reaching the highest value at the SOL-AOL transition (130 m depth, 14.3% of the total *hgcA* abundance). In deeper water layers, *hgcA* abundance decreased to 8.4%–9.9% at 1,000–2,000 m depth. MG-derived *hgcA* genes from *Desulfobacterota* were predominant in both the SOL (28.3%–58.4% of all *hgcA* genes) and the AOL (33.0%–53.8%). The second most abundant MG-derived *hgcA* sequences belonged to *Anaerolineales* and *Phycisphaerae* and predominated in the AOL (representing, respectively, 15.1 ± 6.8% and 6.4 ± 3.1% of all *hgcA* in this layer), while *hgcA* genes from *Kiritimatiellales* and *Bacteroidales* predominated in the SOL (12.9 ± 10.1% and 5.9 ± 5.5%, respectively). At 1,000 and 2,000 m depths, where HS^−^ concentrations were the highest (>350 µM), the proportion of *hgcA* genes from *Verrucomicrobiota* and ^U^*Desulfacyla* decreased, while those from other *Desulfobacterota* and *Chloroflexota* remained dominant (Fig. 5B; Data Sheet S1B). Also, *hgcA* genes from *Firmicutes* represented 1.4%–6.7% of all reads at the SOL-AOL transition (110–130 m), while they were undetected in all the other sampled depths (Data Sheet S1B and D).

### Relationships between environmental gradients, Hg-related variables, and microbial stratification over depth

PCoA analyses showed that the structure of both the 16S rRNA gene-based prokaryotic community (Fig. 3A) and *hgc*^+^ microbial groups (Fig. 3B) clustered similarly with depth (PERMANOVA, *P* < 0.05), according to the three redox zones (OL, SOL, and AOL). This microbial stratification over depth can be related to the observed geochemical gradients of oxygen, HS^−^, and MeHg concentrations, along with other stratified parameters (Table 1).

For the MEDBlack samples , the qPCR-estimated abundances of *hgcA* genes from *Desulfobacterota*, *Firmicutes*, and *Archaea-Chloroflexota* were significantly positively correlated with MeHg and HS^−^ concentrations, and negatively correlated with oxygen concentrations (Spearman rank correlations, *P*-value <0.01; Fig. 3C). Several other environmental parameters also appeared to be stratified and co-varied with oxygen along depth (Table 1).

For station 2 studied in the Phoxy cruise, the correlations between the MG-estimated abundance of *hgcA* genes and environmental conditions depended on the microbial groups (Fig. 3C). The abundances of *hgcA* genes from *Bacteroidota* and *Verrucomicrobiota* significantly increased with oxygen depletion but were not significantly correlated to HS^−^ concentrations. In contrast, *hgc*^+^ *Chloroflexota*, KSB1 Candidate Division, and *Planctomycetota* were positively correlated to HS^−^ concentrations and not significantly related to oxygen concentrations. Finally, *hgcA* genes from *Desulfobacterota* were negatively correlated to oxygen concentrations and positively correlated to HS^−^ concentrations (Fig. 3C). The *hgc*^+^ community clustering in the AOL was characterized by discriminant *hgcA* genes affiliated to *Desulfobacterota* (^U^*Desulfatibia*), *Chloroflexota* (*Anaerolineales*), and *Planctomycetota* (mainly from *Phycisphaerae*), while the *hgc*^+^ community in the SOL was discriminated by *hgcA* genes affiliated to *Kiritimatiellales*, ^U^*Desulfacyla*, *Bacteroidales*, *Desulfobacterales,* and other PVC (Planctomycetota, Verrucomicrobiota, and Chlamydiota) superphylum members (lefse analysis , *P* < 0.05, Fig. S4), thus confirming the oxygen-dependent niche partitioning of potential methylators at a fine taxonomic resolution.

## DISCUSSION

### Coupling methodological approaches to identify bioindicators of Hg methylation in the environment

Our 16S rRNA gene metabarcoding data support previous studies that reported the enrichment of *Desulfobacterota*, *Planctomycetota*, *Acidobacteriota*, and *Firmicutes* with increasing depth, and the restriction of *Nitrospirota*, *Chloroflexota*, and *Verrucomicrobiota* to the anoxic waters of the Black Sea (28, 41). Several of the dominant microbial lineages found in and below the redoxcline include *hgc*^+^ microorganisms. However, studies based on 16S rRNA gene taxonomic classification are often not sufficient to predict the occurrence of Hg methylators, since Hg methylation is a species- (and possibly strain-) specific trait (3). Alternative approaches targeting the *hgc* genes as a biomarker of microbial Hg methylation have been proposed (37, 42, 43). To avoid the biases due to the large polyphyletic diversity of the *hgcA* gene (9), and the overrepresentation of *hgcA* sequences from *Desulfobacterota* in databases of known Hg methylators used for primer design (8, 43), clade-specific qPCR primers have been developed, targeting *hgcA* genes from each of the three dominant Hg-methylating clades, i.e., *Desulfobacterota*, *Archaea*, and *Firmicutes* (37). This approach yielded results similar to metagenome-derived estimates for the identification of Hg methylation biomarkers (42), and is preferable to detect taxa-dependent correlations with environmental and/or functional outcomes. It is important to highlight that the outcomes of different methods should be interpreted cautiously since some discrepancies have been previously reported between, e.g., metagenomics, functional gene sequencing, cloning, qPCR, and 16S metabarcoding (14, 42, 44). Finally, DNA-based approaches should be complemented by gene expression and methylation rates measurements in order to confirm the involvement of potential Hg methylators.

### Geochemical and microbial homogeneity along the Black Sea transect

Here, although qPCR and metagenomic estimates have been applied to samples from two different cruises, the results of the two cruises yield comparable results, including (i) similar stratification pattern of *hgcA* abundance across water depth and environmental conditions, and (ii) similar taxonomic identification and abundance distribution of putative Hg methylators. Even if metagenomic analysis has been carried out only at station 2 during the Phoxy cruise, our results are likely representative for the basin as a whole because of the high horizontal homogeneity of the Black Sea (excluding coastal areas), in terms of microbial community structure (as shown in the present and previous studies) (26) hydrography (27) and geochemistry (25). Here, all the microbial indicators and metrics (i.e., total abundances, abundance of *hgcA*-carriers from different clades, alpha diversity, community structure, and composition) were similar across the east-west transect, as well as the full-depth high-resolution Hg and MeHg concentration profiles. Significant positive correlations were observed between *hgcA* qPCR estimates from the three targeted microbial groups (i.e., *Desulfobacterota*, *Firmicutes*, and *Archaea-Chloroflexota*) and MeHg concentrations. Such correlative links between *hgcA* (gene or transcript) abundance and MeHg concentration and/or formation activity have been previously reported (19, 45, 46), but are not always observed, depending on the ecosystems (47, 48), as documented also for other metabolic processes (49).

### A diverse community of *hgc*^+^ microorganisms is distributed along the redox gradient in the Black Sea

We demonstrate that not only the overall community but also the abundance and diversity of *hgc*^+^ microorganisms vary with the redox gradient in the water column of the Black Sea. The very low proportion of *hgc* genes detected in the oxic layer (and their taxonomic affiliation) is further discussed in Supplemental Information 3.1. Our findings showing a strong relation between oxygen depletion and *hgc*^+^ microorganism abundance are in line with previous studies in marine, brackish, and freshwater environments (15, 17 - 19). We provide the first evidence for microbial potential to produce MeHg in the anoxic sulfidic waters of the Black Sea, as previously suggested by a modeling approach based on geochemical data (25). This disagrees with earlier studies where persistently high sulfide concentrations were believed to inhibit Hg methylation in the AOL of the Black Sea (24) and other systems (50, 51). Similar to our results, maximal methylation activity was also reported under sulfidic conditions in stratified brackish water (19) and a meromictic lake (52). The dual role of sulfide in Hg methylation is complex and still uncertain (51, 53).

*Desulfobacterota* members exhibit the highest diversity and abundances of *hgcA* genes in the Black Sea water column. The presence of *Desulfobacterota* in the anoxic water layer of the Black Sea can be explained by their metabolisms as sulfate reducers, iron reducers, and/or fermentative syntrophic in oxygen-deficient environments (54). Sulfate reduction has been previously reported in the Black Sea anoxic water (30, 55) and is also supported by our high HS^−^ concentrations in the AOL. For decades, *Desulfobacterota* members have been confirmed to be capable of Hg methylation and/or carrying *hgc* genes (3, 11). *Desulfobacterota* have also previously been identified as prevalent *hgc*^+^ microorganisms in many anoxic marine waters (17 - 19) and sediments (13, 56). In our work, certain *hgcA* genes belong to MAGs previously reconstructed from the Phoxy metagenomes (30). Among them, the *hgc*^+^ MAGs ^U^*Desulfatibia profunda* NIOZ-UU30 and ^U^*Desulfacyla* NIOZ-UU19, enriched in the anoxic waters, were described as probably having a strict sulfate-reducing lifestyle. By contrast, the *hgc*^+^ sulfate reducers ^U^*Desulfacyla euxinica* NIOZ-UU27,^U^*Desulfatibia vada* NIOZ-UU17, and ^U^*Desulfobacula maris* NIOZ-UU16, which were abundant in the suboxic waters and dominant at the top of the anoxic waters, feature a more flexible potential metabolism, with the ability to gain energy from the reduction of diverse electron acceptors (S^0^, thiosulfate, tetrathionate, nitrate, nitrite), possibly including oxygen respiration (30). The MAG of the SRB ^U^*Desulfobia pelagia* NIOZ-UU47, for which *hgcA* genes were found between 105 and 250 m, seems to have the ability to fix nitrogen. Altogether, these results show that *Desulfobacterota*-mediated MeHg formation in the Black Sea may be coupled to sulfate reduction but also to other metabolic pathways depending on the redox niche.

Certain *Anaerolineales* (*Chloroflexota*) are also part of the dominant *hgcA*-carriers in the suboxic and anoxic waters of the Black Sea including the MAGs NIOZ-UU11 and NIOZ-UU52. *Anaerolineales* are fermenters, in possible syntrophic association with methanogens (57). *Anaerolineales* MAGs were previously identified as highly abundant in the Black Sea, where some of them carried sulfate reduction genes (28). Although there is still limited genomic information about members of this group, a *hgc*^+^ MAG *Anaerolineales* representative (BSW_bin111) was detected in the anoxic brackish water of the Baltic Sea (19). In addition, *Phycisphaerae* (*Planctomycetota*), *Kiritimatiellales* (*Verrucomicrobiota*), and *Bacteroidales* (*Bacteroidota*) were identified as major potential Hg methylators in the oxygen-deficient waters of the Black Sea. Among these new, underappreciated, fermentative *hgcA* carriers, the *Bacteroidota* MAG NIOZ-UU65 was equipped with polysulfide reductase genes (30). *Phycisphaerales* have been identified in the euxinic waters of the Cariaco basin as attached to particles (58) and in the Black Sea (59) as potential degraders of newly formed OM not linked to the redox gradient (60), and some type strains seemed capable of nitrate reduction (61). *Phycisphaerales* have been reported as minor putative Hg methylator in marine sediments (13), salt marsh, and freshwater sediment (43). Culture and single-cell genomics of *Kiritimatiellales* representatives indicated a fermentative lifestyle, with the capacity for degradation of complex and recalcitrant polysaccharides and glycoproteins, as well as hydrolysis of sulfate esters, with wide oxygen and salinity tolerance, and a preference for biofilm habitats (62 - 65). *Kiritimatiellales* were previously identified by metagenomic approaches among the most prominent putative Hg methylators in sediment and anoxic water of eutrophic sulfate-rich freshwater lakes (14, 15) and in the anoxic brackish water of the Baltic Sea (19). Overall, fermenters from *Anaerolineales*, *Phycisphaerae*, *Kiritimatiellales*, and *Bacteroidales* have been rarely reported as putative Hg methylators, usually at low abundance in ecosystems different from the present one. To our knowledge, this is the first report of their substantial joint contribution to Hg methylation, conforming a unique assemblage in a permanently stratified marine ecosystem. Among these diverse Hg methylators candidates found in the Black Sea suboxic/anoxic waters, some lineages (*Anaerolineales* and *Kiritimatiellales*) are common to other euxinic basins (17, 19), suggesting that they may be part of the important mediators of Hg methylation in permanently or not permanently anoxic water from marine and/or brackish environments with high sulfide concentrations. However, it is unclear if this diversity is a common trait of (not euxinic) OMZ, where Hg methylators have been rarely detected (10). Further studies should compare the *hgc^+^* MAGs identified here and in similar oxygen-depleted marine systems, to explore the metabolic versatility of Hg-methylating microorganisms and their environmental drivers.

Finally, our results suggest that the qPCR estimates of *hgcA* genes identified by primers primarily designed to target *Archaea* are overestimated. This is supported by cloning-sequencing of PCR products obtained using *Archaea*-specific *hgcA* qPCR primers, showing that some amplicon sequences group with *Chloroflexota* rather than *Archaea* (Supplemental Information 1.4, Fig. S2). Additionally, our metagenome analyses consistently show that *hgc*^+^ *Chloroflexota* are major putative Hg methylation contributors, especially in the AOL, while *Archaea*-assigned *hgcA* are rare and not abundant (Data Sheet S1B and D). Recently, an analysis of publicly available MAGs revealed high similarities of *hgc* genes from *Euryarchaeota* and *Chloroflexota*, potentially due to horizontal gene transfers (9). Altogether, these results suggest that the *Archaea-hgcA* primers from Christensen et al. (37) are less specific than initially thought and likely include *hgcA* genes from other clades such as *Chloroflexota*.

### Conclusion

Oxygen-limited and anoxic areas are spreading in the coastal and offshore ocean, implying modifications of biogeochemical cycles. The Black Sea offers a unique opportunity to study the effect of oxygen gradients on biogeochemical cycles such as Hg transformations. Our findings highlight that a unique combination of diverse dominant *hgc*^+^ microbes can coexist and jointly contribute to MeHg production in marine environments, with niche partitioning according to the redox gradient. We identified members of *Desulfobacterota*, *Chloroflexota*, *Verrucomicrobiota*, *Planctomycetota,* and *Bacteroidota* as the main actors of Hg methylation in the Black Sea water column. The microbial communities, including putative Hg methylators, were horizontally homogeneous across the Black Sea, but vertically stratified. The abundance of *hgcA* genes increased with depth, being positively correlated with MeHg concentration and negatively with oxygen concentration. Our DNA-based results support that Hg methylation potentially occurs predominantly in the anoxic waters of the Black Sea, which should be further confirmed by measuring gene expression and/or methylation rates. Microorganisms harboring *hgc*^+^ were dominated by *Desulfobacterota*, followed by a high diversity of previously less recognized Hg methylators belonging to *Phycisphaerae*, *Kiritimatiellales,* and *Anaerolineales*. The strong environmental gradients across the Black Sea water column affect the microbial community composition, resulting in a partitioning of Hg-methylating microorganisms, and their associated metabolic pathways, differing across redox niches. Our results, robustly validated by two different methodological approaches (qPCR and metagenomics), were consistent with measured environmental parameters, including MeHg concentration. By identifying marine anoxic niches as a primary MeHg source, this study is of major relevance in the context of global warming and anthropogenic activity which currently result in enhanced seawater deoxygenation and global expansion of anoxic zones.

## Data Availability

Raw sequences have been deposited at NCBI GenBank, SRA database, under the BioProject accession number PRJNA895066.

## SUPPLEMENTAL MATERIAL

Supplemental Information msystems.00537-23-s0001.docx Table S1, supplemental text, and Figure S1 to S4.

Data Sheet msystems.00537-23-s0002.xlsx Environmental and molecular data from both cruises, OTU abundance table and taxonomy, *hgcA* and *hgcB* genes found in Phoxy cruise metagenomes, and community composition*.*

ASM does not own the copyrights to Supplemental Material that may be linked to, or accessed through, an article. The authors have granted ASM a non-exclusive, world-wide license to publish the Supplemental Material files. Please contact the corresponding author directly for reuse.
