# Supplementary material for: Redox gradient shapes the abundance and diversity of mercury-methylating microorganisms along the water column of the Black Sea
Source: mSystems. 2023 Aug 14;8(4):e00537-23. doi: 10.1128/msystems.00537-23 (PMC10469668; doi:10.1128/msystems.00537-23)
Supplement: Supplemental Information — Table S1, supplemental text, and Figure S1 to S4. [file msystems.00537-23-s0001.docx]

Supplementary Information for:

**Redox gradient shapes the abundance and diversity of mercury-methylating microorganisms along the water column of the Black Sea**

Léa Cabrol, Eric Capo, Daan M. van Vliet, F. A. Bastiaan von Meijenfeldt, Stefan Bertilsson, Laura Villanueva, Irene Sánchez-Andrea, Erik Björn, Andrea G. Bravo, Lars-Eric Heimbürger-Boavida

**Content of Supporting Information:**

[Supplementary Table 1: PCR and qPCR conditions 2](#_Toc130307041)

[1. Supplementary Material and Methods 3](#_Toc130307042)

[**1.1.** **Sampling and analysis of mercury species** 3](#_Toc130307043)

[**1.2.** **Bioinformatics treatment of 16S rRNA amplicon sequences** 3](#_Toc130307044)

[**1.3.** **Clade-specific *hgcA* gene qPCR-estimates** 3](#_Toc130307045)

[**1.4.** **Cloning-sequencing of *hgcA* sequences from Archaea** 4](#_Toc130307046)

[**1.5.** **Metagenomic-estimates of *hgcA* genes** 5](#_Toc130307047)

[2. Supplementary Results 6](#_Toc130307048)

[**2.1.** **Prokaryotic community** 6](#_Toc130307049)

[3. Supplementary Discussion 6](#_Toc130307050)

[**3.1.** **Low abundance of *hgc*+ microorganisms in the oxic water of the Black Sea** 6](#_Toc130307051)

[Supplementary Figure S1: Mercury profiles 8](#_Toc130307052)

[Supplementary Figure S2: Phylogeny of Archaea-qPCR products 9](#_Toc130307053)

[Supplementary Figure S3: Taxonomic composition of all replicates 10](#_Toc130307054)

[Supplementary Figure S4: Discriminant *hgcA* genes along depth 11](#_Toc130307055)

[Description of Datasheet 1 12](#_Toc130307056)

[Additional references for Supplementary Information: 13](#_Toc130307057)

# **Supplementary Table 1: PCR and qPCR conditions**

Experimental conditions for Bacteria and Archaea 16S qPCR, clade-specific *hgcA* qPCR, and 16S rRNA gene PCR for metabarcoding sequencing. qPCR reactions were carried out on CFX96 thermocycler (Bio-Rad Laboratories, Hercules, US), PCR reactions were carried out on T100 Thermocycler, BioRad (Hercules, US). All reactions were run with 10-fold diluted DNA extracts. Standard curves for *Bacteria* and *Archaea* quantification were generated from 10^1^ to 10^8^ copies of 16S rRNA gene template per µL, using a pGEMT plasmid containing, respectively, a gammaproteobacterial (*Pseudomonas stutzeri* SLG510A3-8) or euryarchaeaotal SSU rRNA gene fragment (clone Arch508, accession number HE647171), with amplification efficiency ranging from 86 to 95%.

| **Method** | **Group** | **Primers id** | **Primers sequence** | **Final primer concentration (µM)** | **Amplification program** | **Nb of cycles** | **Amplicon length (bp)** | **Reference** |
| --- | --- | --- | --- | --- | --- | --- | --- | --- |
| **qPCR** | Total Bacteria 16S | GML5F | 5’- GCCTACGGGAGGCAGCAG-3’ | 0.25 | 95°C 2 min | 40 | 172 | Muyzer et al. (1993), Takai and Horikoshi (2000) |
|  |  |  |  |  | 95°C 15s |  |  |  |
|  |  | Univ516R | 5’- GTDTTACCGCGGCKGCTGRCA -3’ | 0.25 | 55°C 20s |  |  |  |
|  |  |  |  |  | 72°C 30s |  |  |  |
|  | Total Archaea 16S | Arc344F | 5’-ACGGGGYGCAGCAGGCGCGA-3’ | 0.25 | 95°C 2 min | 40 | 172 | Takai and Horikoshi, 2000 |
|  |  |  |  |  | 95°C 15s |  |  |  |
|  |  | Univ516R | 5’-GTDTTACCGCGGCKGCTGRCA-3’ | 0.25 | 62°C 20s |  |  |  |
|  |  |  |  |  | 72°C 30s |  |  |  |
|  | Desulfobacterota-specific hgcA | hgcA-Delta-F hgcA-Delta-R | 5’-GCCAACTACAAGMTGASCTWC-3’ 5’-CCSGCNGCRCACCAGACRTT-3’ | 0.4 0.4 | 95°C 3 min | 45 | 107 | Christensen et al., 2016 |
|  |  |  |  |  | 95°C 30 s |  |  |  |
|  |  |  |  |  | 62.5°C 60 s |  |  |  |
|  | Firmicutes-specific hgcA | hgcA-Firm-F | 5’-TGGDCCGGTDARAGCWAARGATA-3’ | 0.25 | 95°C 3 min | 40 | 167 | Christensen et al., 2016 |
|  |  |  |  |  | 95°C 10 s |  |  |  |
|  |  | hgcA-Firm-R | 5’-AAAAGAGHAYBCCAAAAATCA-3’ | 0.25 | 47°C 10 s |  |  |  |
|  |  |  |  |  | 58°C 60 s |  |  |  |
|  | Archaea-specific hgcA | hgcA-Arch-F | 5’-AAYTAYWCNCTSAGYTTYGAYGC-3’ | 0.5 | 95°C 3 min | 40 | 125 | Christensen et al., 2016 |
|  |  |  |  |  | 95°C 30 s |  |  |  |
|  |  | hgcA-Arch-R | 5’-TCDGTCCCRAABGTSCCYTT-3’ | 0.25 | 50°C 10 s |  |  |  |
|  |  |  |  |  | 55°C 60 s |  |  |  |
| **PCR for 16S amplicon sequencing** | Total Bacteria and Archaea | 515F | 5’-GTGYCAGCMGCCGCGGTA-3’ | 0.25 | 98°C 30s | 30 | 380 | Wang and Qian, 2009 |
|  |  |  |  |  | 98°C 5s |  |  |  |
|  |  | 928R | 5’-CCCCGYCAATTCMTTTRAGT-3’ | 0.25 | 72°C 15s |  |  |  |
|  |  |  |  |  | 72°C 5 min |  |  |  |

# **1. Supplementary Material and Methods**

## **Sampling and analysis of mercury species**

In the MEDBlack cruise, high-resolution vertical water profiles for geochemistry were obtained using a titanium ultraclean CTD frame^30^ equipped with 24 x 24L PVDF samplers.

The dissolved concentrations of total Hg and MeHg (sum of mono- and di-MeHg) were measured according to previous protocols^23^. Briefly, tHg was measured following the USEPA 1631 method modified by Heimbürger et al., 2015, using ultra-clean BrCl and a custom-made semi-automatic single gold trap setup coupled to a cold vapor atomic fluorescence spectrometer. MeHgD was analyzed via isotope dilution (addition of enriched spikes of ^199^iHg and ^201^MeHg) and extraction in organic solvent, using a high-sensitivity coupled gas chromatograph-sector field ICP-MS (GC-SF-ICP-MS) (Heimbürger et al., 2015). The method was adapted to anoxic samples by oxygenating the samples after the spike addition.

## **Bioinformatics treatment of 16S rRNA amplicon sequences**

Sequence reads were demultiplexed, and paired-end reads merged with a maximum of 10% mismatch in the overlapped region, by FLASH (Fast Length Adjustment of Short reads) (Magoč and Salzberg, 2011). Raw sequence reads in the FastQ format were analyzed on the Galaxy bioinformatics platform (http://sigenae-workbench.toulouse.inra.fr) through the FROGS (Find Rapidly OTU with Galaxy Solution) pipeline, version 3.2.3 (Escudié et al., 2018). 16S rRNA sequences were denoised and dereplicated. Sequences with a length between 350 and 450 nucleotides were selected. Sequences without both primers and with ambiguous bases were removed. In the remaining sequences, Cutadapt (Martin, 2011) was used to search and trim the primer sequences (< 10% mismatches allowed). Operational Taxonomic Units (OTUs) were defined by sequence clustering, using the iterative growth process of the SWARM algorithm v3.2.3 (Mahé et al., 2021) with aggregative distance d=1, as recommended in the “—fastidious” option. After aggregation, clustering was refined by checking that the abundances are decreasing along the connections away from the seed; otherwise, the connection was cut. A representative seed sequence was chosen for each OTU. Chimeras were detected and removed using the VSEARCH algorithm (v.1.9, <https://github.com/torognes/vsearch>). OTU sequences were filtered to remove OTUs with relative abundance lower than 0.005 %, as previously recommended (Bokulich et al., 2013). Taxonomic affiliation of each OTU was performed using the BLAST tool implemented in Galaxy against the non-redundant 16S database from SILVA (version 138.1).

A total of 330.412 raw DNA sequences, reduced to 239.360 clean sequences (ranging from 9.049 to 31.590 per sample), were obtained and grouped into 1.221 OTUs (Datasheet 1C).

## **Clade-specific *hgcA* gene qPCR-estimates**

We used the clade-specific degenerate qPCR primers developed by (Christensen et al., 2016) in order to target the hgcA gene for each of the three dominant Hg-methylating clades, i.e., Desulfobacterota, Firmicutes, and Archaea. Primer sequences and concentrations as well as expected amplicon size and qPCR conditions are provided in Supplementary Table S1. qPCR reactions were carried out on CFX96 thermocycler (Bio-Rad Laboratories, Hercules, CA, US), using Takyon No Rox SYBR 2X Master Mix (Eurogentec, Seraing, Belgium). The dilution factor of raw DNA was optimized, resulting in the selection of a 10-fold dilution for all samples in order to avoid inhibition problems. For each clade, the qPCR conditions were evaluated and optimized. The qPCR efficiency was compared at the hybridization temperature suggested in Christensen et al (2016) and at two other temperatures (i.e., 2°C lower and 2°C higher). For Firmicutes and Archaea, the 3-step optimized qPCR program was highly similar to that of Christensen et al (2016), for Desulfobacterota the 2-step qPCR program was slightly modified, e.g., by decreasing hybridization temperature and increasing primer concentration and annealing step, as shown in Supplementary Table S1. The standards used for Desulfobacterota and Firmicutes hgcA quantification were, respectively, fragments of *hgcA* gene from *Desulfovibrio desulfuricans* ND132 ATCC7757 and *Desulfosporosinus yougiae* JW/YJLB18 pure cultures, ligated in pGEM-T easy vector (Promega, Madison, WI, US) and cloned in E. coli JM109. The cloned plasmids were extracted, purified and sequenced (GATC Biotech, Constance, Germany) to confirm affiliation to the correct target clade. The standard used for Archaea hgcA quantification was a synthetic fragment of the *hgcA* gene of *Methanomassilicoccus luminyensis* (longer than the fragment targeted by qPCR) inserted in pUC57 plasmid (Eurogentec, Seraing, Belgium). The melting curves and agarose gel electrophoresis confirmed that a unique PCR product of correct size was amplified for Desulfobacterota and Firmicutes. The serial dilutions (10^1^ to 10^8^ copies µL-1) of *hgcA* standards provided qPCR efficiencies of 87, 94 and 81 %, for the hgcA quantification of respectively Desulfobacterota, Firmicutes and Archaea. The detection limits were <10^2^, 10^1^ and 10^3^ copies µL-1, respectively, for Desulfobacterota, Firmicutes, and Archaea hgcA quantification. Results are displayed in Datasheet 1A.

## **Cloning-sequencing of *hgcA* sequences from Archaea**

Archaea-*hgcA* amplicons were obtained by PCR with GoTaq G2 HotStart DNA Polymerase (Promega) following the same procedure as for qPCR (Supplementary Table S1). PCR products were purified (QIAquick PCR Purification Kit, Qiagen) and ligated in pGEM-T Easy vector (Promega) using 3 µL of purified PCR product, 5 µL of 2X buffer, 1 µL of 50 ng µL^-1^ plasmid and 1 µL of T4 DNA ligase at 3 U µL^-1^ (Promega) at 4°C overnight. Transformation of 50 µL competent E. coli JM109 cells (Promega) with 10 µL of ligation product plasmids was carried out by heat-shock according to the provider’s instructions, incubated in SOC medium and plated on LB-agar petri dishes with 100 µg mL^-1^ ampicillin, 100 µL of 100 mM filtered IPTG and 75 µL of 50 mg mL^-1^ S-Gal. After overnight incubation, white colonies were picked, correct plasmid insertion was verified by PCR with M13 primers, and 92 selected colonies of interest were cultured in LB-ampicillin liquid medium overnight. Plasmid DNA was extracted from cell pellets with the Qiaprep Spin Miniprep Kit (Qiagen) and sequenced by GATC (Eurofins).

Nucleotide sequences were aligned with Muscle (MEGAX software). Obtained DNA sequences were translated into amino acid sequences and compared with hgcA sequences from the Hg-MATE database and from the 15 metagenomes obtained in the Phoxy cruise (Section 2.6).

For hgcA sequences with the conserved amino acid motif NVWCAGK obtained using Archaea hgcA-specific qPCR primers, 12 and 5 unique sequences with 34 and 21 copies were obtained from AOL samples at stations 5 and 9, respectively (Fig. S2). Most of the hgcA sequences clustered with Phoxy cruise MG-derived hgcA sequences and were grouped in two clusters: (i) cluster 1 related to Euryarchaeota (Theionarchaea), (ii) cluster 2 related to a mixed pool of hgcA sequences from both Euryarchaeota and Chloroflexota; while two other sequences were identified as Chloroflexota (Anaerolineales, F5B 011) and as mixed (F9B 011). Thus, from there on, *hgcA* qPCR estimates from *Archaea* will be referred to *hgcA* qPCR estimates from *Archaea-Chloroflexota* in the continuation of this study.

## **Metagenomic-estimates of *hgcA* genes**

After sequencing on 2*250 bp PE Illumina MiSeq, quality control of metagenomes was performed with FastQC v0.11.3 and reads with uncalled bases and remaining TruSeq adapters were removed with Flexbar v2.5 (Dodt et al., 2012), keeping the longer side of the read with the ‘--ae any’ flag. All reads were cross-assembled with SPAdes v3.8.0 in ‘--meta’ mode (Nurk et al., 2017), with read error correction turned on. BWA-MEM v0.7.12-r1039 (Li, 2013) was used to map the forward and reverse reads from individual samples to the cross-assembled scaffolds.

In addition to hgcA detection described in the manuscript, we also looked for unique conserved motifs from *hgcB* genes (C(M/I)EC(G/S)(A/G)C) and performed a manual inspection of the presence of *hgcB* genes. Certain *hgcA* genes were found side-by-side with *hgcB* genes on the same contig (Datasheet 1D). Because *hgcB* genes are not always found paired to *hgcA* genes in genomes, only counts from *hgcA* genes were considered in our estimates of the distribution of *hgc*^+^ microorganisms at different water depths.

After having detected the *hgc* homologs in metagenomes (as described in the main manuscript), they were taxonomically affiliated by a phylogenetic analysis, using the reference package ‘hgcA’ from Hg-MATE database v1 (Gionfriddo et al., 2021). Briefly, the predicted amino acid sequences from genes identified as putative *hgcA* gene were (i) compiled in a FASTA file, (ii) aligned to Stockholm formatted alignment of HgcA sequences from the reference package with the function *hmmalign* from HMMER 3.2.1 (iii) placed onto the HgcA reference tree with the function *pplacer* and (iv) classified using the functions *rppr* and *guppy_classify* from the program pplacer (Matsen et al., 2010).

We followed the recommendations of Capo et al. 2022 (*Molecular Ecology Resource*) in their standardized protocol for the detection, identification and quantification of *hgc* genes from metagenomes, which is important for comparability purpose with previous and future studies. We thus used the reference phylogenetic tree provided by the Hg-MATE database (RAxML_bipartitions.Hg-MATE-Db.v1.ISOCELMAG-HgcA-Full-ML-100bs-tree-rooted) built by RAxML using *hgcA* sequences with genomic identifying information from (i) pure culture/environmental microbial isolates (204 sequences), (ii) single-cell genome sequences (29 sequences), and (iii) metagenome-assembled genomes (787 sequences). This tree was rooted with distant paralogs and its statistical robustness was validated by bootstrapping. Then a simplified tree of was built where clades were collapsed by the dominant monophyletic group, to improve visualization. This simplified unrooted tree is the one presented in the methodological protocol proposed in Capo et al., 2022, and also the one we used in our study, because it is the most complete to date.

The *hgcA* gene found in MAG NIOZ-UU65 (Bacteroidota) is identified as a Bacteroidales with Hg-MATE phylogeny and is thus described as Bacteroidales in the whole text.

# **Supplementary Results**

## **Prokaryotic community**

The lowest archaeal abundances determined by qPCR were observed in the OL (0.3% of the prokaryotes on average), while higher abundances were observed in the AOL and the SOL, representing on average 9.7 and 8.3% of total prokaryotes, respectively. qPCR-based archaeal abundances were broadly consistent with estimations froml16S rRNA gene amplicon sequencing of the total prokaryotic community, where *Archaea* represented 5.3 ± 2.5% of the community in the AOL, 1.6 ± 1.0% in the SOL and 0.24 ± 0.3% in the OL.

For all stations, OL communities were dominated by *Cyanobacteria* representing 70% of prokaryotes, followed by *Planctomycetota* and *Bacteroidota* accounting for less than 9% (Fig 2D, Fig S3. Datasheet 1C&1E). In contrast, SOL and AOL were dominated by *Planctomycetota* (29-47% of the prokaryotes), *Bacteroidota* (13-15%) and *Desulfobacterota* (7-9%). *Desulfobacterota* were mainly represented by members of genera *Desulfatiglans*, *Desufloconvexum* and *Desulfobacula* and families *Desulfurivibrionaceae*, *Desulfosarcinaceae* and *Desulfobulbaceae*. Finally, *Chloroflexota* were dominant in AOL (11%) but not (< 2%) in SOL communities. Shannon diversity indexes were homogeneous along the west-east transect (ANOVA, p = 0.16) but increased significantly over depth (ANOVA, p < 0.001), reaching the highest values in AOL (Fig. 2C, Datasheet 1A). The lower diversity in OL might be due to the overabundance of *Cyanobacteria*.

# **Supplementary Discussion**

## **Low abundance of *hgc*+ microorganisms in the oxic water of the Black Sea**

Beside the most abundant *hgc*+ microorganisms predominating in the suboxic and anoxic layers (as discussed in the main manuscript), some *hgc* genes were detected by qPCR and MG in the oxic layer, at very low proportion (0.07 % of the total *hgc* coverage values in the metagenomes from the whole water column), suggesting that Hg methylation is not a significant process in the oxic waters of the Black Sea. These genes belong to members of microbial phyla mostly considered as anaerobic i.e., Desulfobacterota (Waite et al., 2020) Chloroflexota (Anaerolineales) (Yamada et al., 2006), Verrucomicrobiota (Kiritimatiellales) (van Vliet et al., 2020; Van Vliet et al., 2019), Planctomycetota (Fuerst, 2017; Wiegand et al., 2018), Bacteroidota (Hudson and Egan, 2022) and Spirochaetota (Lee et al., 2015; Veldkamp, 1960). Their presence as free-living organisms in the oxic later is unexpected but consistent with the potential role of settling particles as anaerobic niches within oxic water zones (Bianchi et al., 2018; Gallorini and Loizeau, 2022; Gascón Díez et al., 2016). As an example, Capo et al. (2020) showed that metagenomes from settling particles exhibited high amounts of *hgcA* genes in the water column of the Baltic Sea, with *hgc*^+^ Spirochaetes being dominant especially in metagenomes obtained from 0.3 µM filters that retained particulate matter present in the water column. Potential ‘oxic’ Hg-methylation in the oxic subsurface waters does not appear to be a significant process in the Black Sea, contrary to what had been suggested in the East China Sea (Tada et al., 2020) or in the global ocean (Villar et al., 2020). In these two studies, oxic Hg methylation was attributed to *Nitrospina* which is completely different from our results. Note that there are still uncertainties about the fact that *Nitrospina* carry “true” *hgcA* genes, and its methylation capacity has never been demonstrated experimentally. The absence of detectable *hgcA* genes has also been reported in the oxygenated pelagic Arctic Ocean seawater (Bowman et al., 2020).

# **Supplementary Figure S1: Mercury profiles**

**
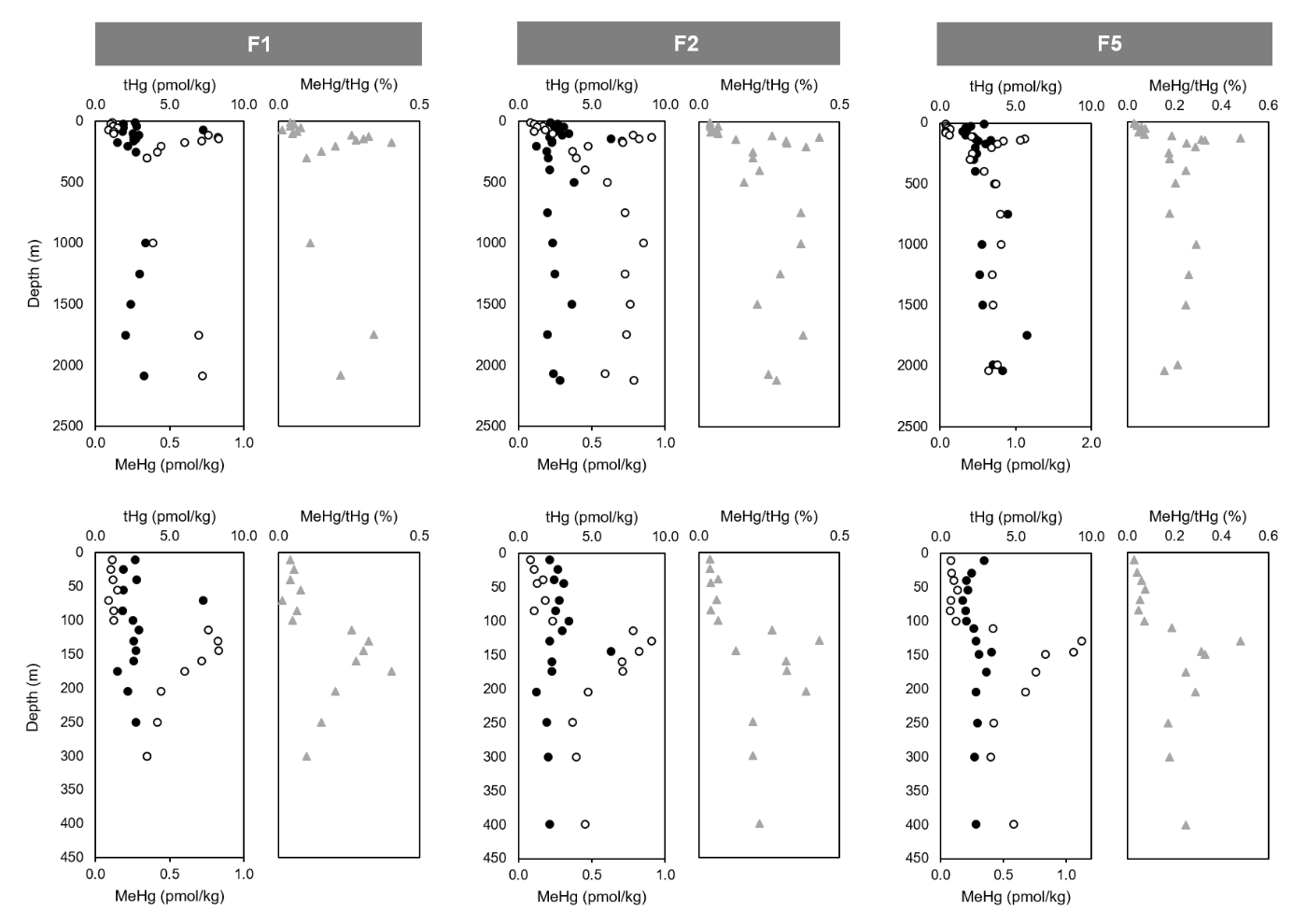
**

**Figure S1.** High resolution depth profiles of total Hg (black dots), MeHg (white dots), and MeHg/Hg ratio (triangles), measured at Stations F1, F2, and F5 during the MedBlack cruise. The top plots show the full depth-profile, the bottom plots are a zoom from 0 to 450 m deep.

# **Supplementary Figure S2: Phylogeny of Archaea-qPCR products**

**Figure S2.** Phylogenetic tree of the cloned *hgcA* genes recovered from the PCR products obtained with Archaea-specific *hgcA* primers from Christensen et al. (2016).

# **Supplementary Figure S3: Taxonomic composition of all replicates**


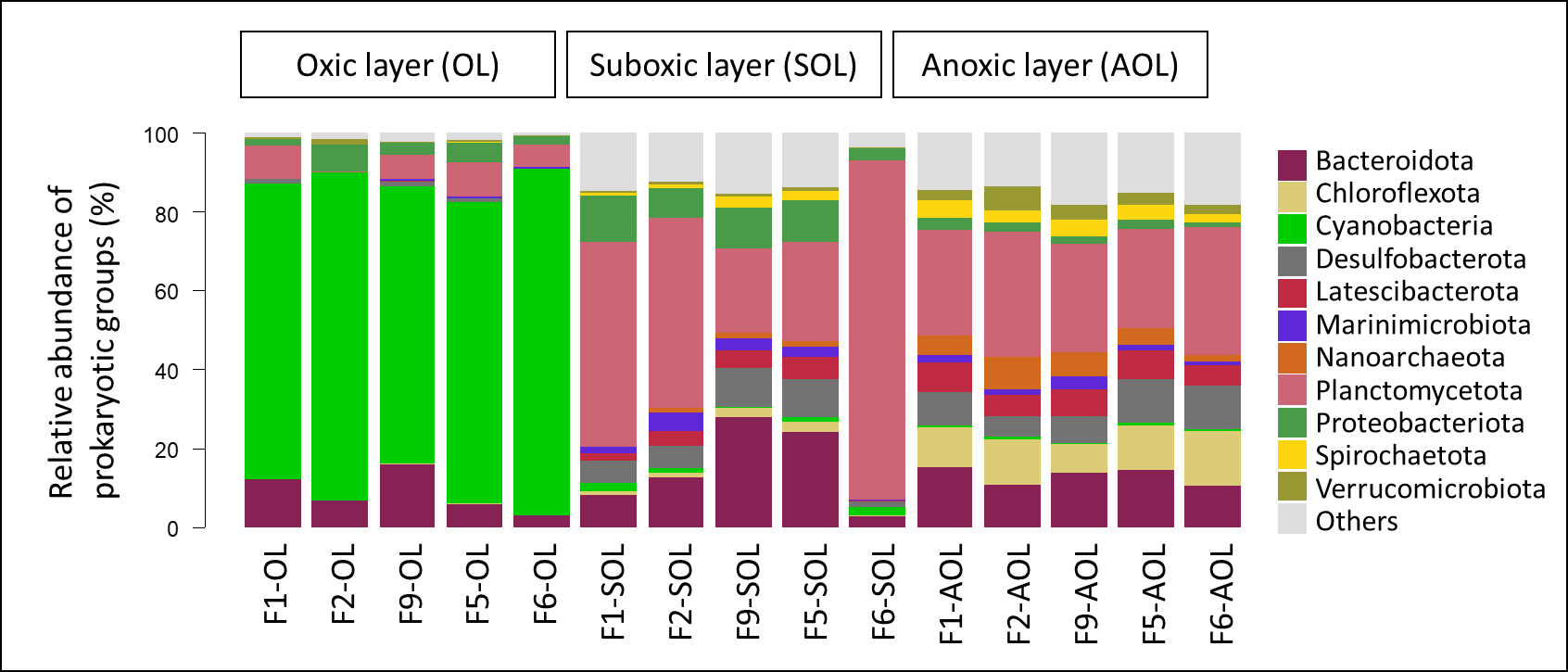


**Figure S3.** Taxonomic composition of the prokaryotic community at the phylum level. The 15 most abundant phyla are represented. Sampling stations along the west-to-east transect are indicated by F1, F2, F9, F5, F6, as shown on Figure 1. Sampling depth along the redox gradient is indicated by OL (oxic layer), SOL (suboxic layer) and AOL (anoxic layer).

# **Supplementary Figure S4: Discriminant *hgcA* genes along depth**

**
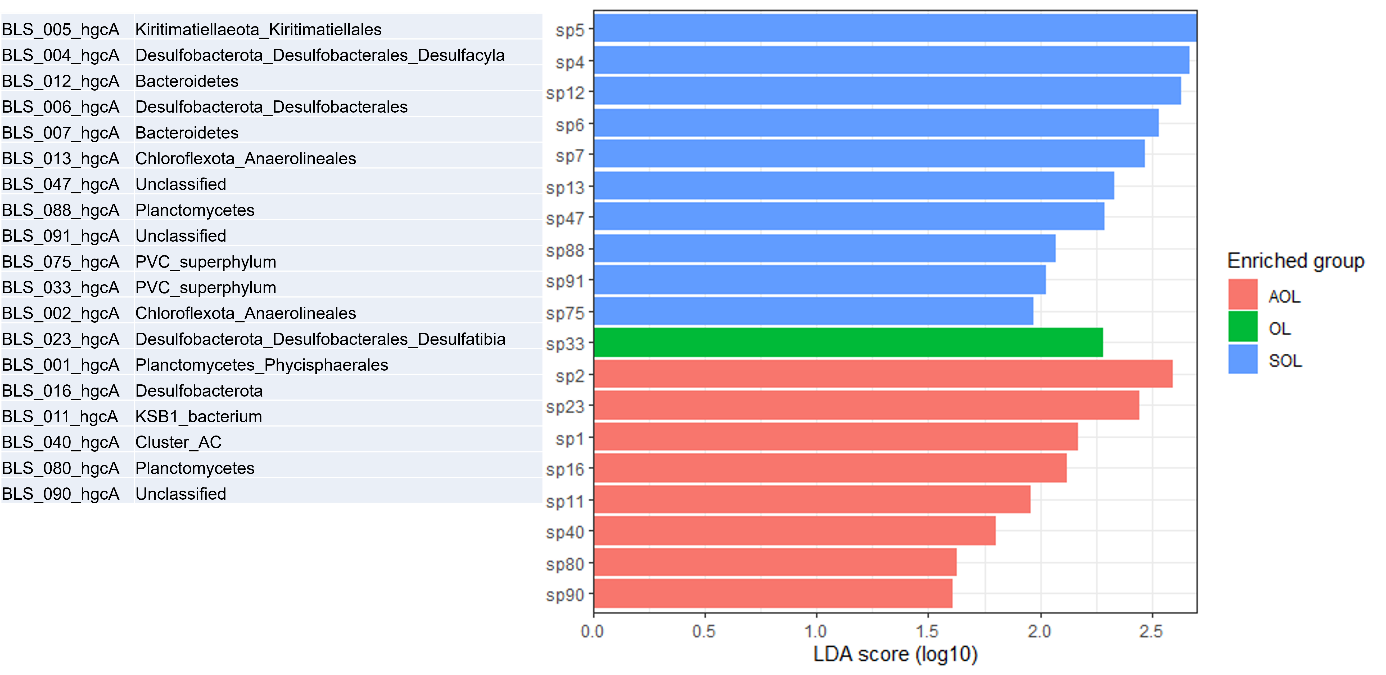
**

**Figure S4.** Identification of discriminant *hgcA* genes related to the clustering of *hgc*^+^ communities along the depth gradient in the three previously defined layers (OL, SOL, AOL), by LDA analysis (run_lefse function, 100 permutations, p value < 0.05).

# **Description of Datasheet 1**

The supplementary Excel spreadsheet joined to this study provides all the produced data in this study, together with some available data from previous studies:

**Datasheet 1A:** Environmental and molecular data produced using samples from MEDBlack cruise, including dissolved concentrations of oxygen, nutrients, mercury and methylmercury; bacterial and archaeal 16S rRNA gene abundances (qPCR), clade-specific hgcA gene abundance (qPCR), diversity indexes (16S amplicon metabarcoding).

**Datasheet 1B:** Environmental and molecular data produced using samples from Phoxy cruise, including dissolved concentrations of oxygen and nutrients; metagenome-estimated hgcA abundances affiliated to key microbial lineages.

**Datasheet 1C:** OTU abundance table and taxonomy, obtained from 16S metabarcoding.

**Datasheet 1D:** Identification, abundance and taxonomic affiliation of hgcA and hgcB genes found in Phoxy cruise metagenomes.

**Datasheet 1E:** Community composition aggregated at the phylum level and averaged per water layer (OL, SOL and AOL).

# **Additional references for Supplementary Information:**

Bianchi, D., Weber, T.S., Kiko, R., Deutsch, C., 2018. Global niche of marine anaerobic metabolisms expanded by particle microenvironments. Nat. Geosci. 11, 263–268. https://doi.org/10.1038/s41561-018-0081-0

Bokulich, N.A., Subramanian, S., Faith, J.J., Gevers, D., Gordon, J.I., Knight, R., Mills, D.A., Caporaso, J.G., 2013. Quality-filtering vastly improves diversity estimates from Illumina amplicon sequencing. Nat. Methods 10, 57–59. https://doi.org/10.1038/nmeth.2276

Bowman, K. L., Collins, R. E., Agather, A. M., Lamborg, C. H., Hammerschmidt, C. R., Kaul, D., ... & Elias, D. A. (2020). Distribution of mercury‐cycling genes in the Arctic and equatorial Pacific Oceans and their relationship to mercury speciation. *Limnology and Oceanography*, *65*, S310-S320.

Christensen, G.A., Wymore, A.M., King, A.J., Podar, M., Hurt, R.A., Santillan, E.U., Soren, A., Brandt, C.C., Brown, S.D., Palumbo, A. V., Wall, J.D., Gilmour, C.C., Elias, D.A., 2016. Development and validation of broad-range qualitative and clade-specific quantitative molecular probes for assessing mercury methylation in the environment. Appl. Environ. Microbiol. 82, 6068–6078. https://doi.org/10.1128/AEM.01271-16

Dodt, M., Roehr, J.T., Ahmed, R., Dieterich, C., 2012. FLEXBAR-flexible barcode and adapter processing for next-generation sequencing platforms. Biology (Basel). 1, 895–905. https://doi.org/10.3390/biology1030895

Escudié, F., Auer, L., Bernard, M., Mariadassou, M., Cauquil, L., Vidal, K., Maman, S., Hernandez-Raquet, G., Combes, S., Pascal, G., 2018. FROGS: Find, Rapidly, OTUs with Galaxy Solution. Bioinformatics 34, 1287–1294. https://doi.org/10.1093/bioinformatics/btx791

Fuerst, J.A., 2017. Planctomycetes-New Models for Microbial Cells and Activities, Microbial Resources: From Functional Existence in Nature to Applications. Elsevier Inc. https://doi.org/10.1016/B978-0-12-804765-1.00001-1

Gallorini, A., Loizeau, J.L., 2022. Lake snow as a mercury methylation micro-environment in the oxic water column of a deep peri-alpine lake. Chemosphere 299, 134306. https://doi.org/10.1016/j.chemosphere.2022.134306

Gascón Díez, E., Loizeau, J.L., Cosio, C., Bouchet, S., Adatte, T., Amouroux, D., Bravo, A.G., 2016. Role of Settling Particles on Mercury Methylation in the Oxic Water Column of Freshwater Systems. Environ. Sci. Technol. 50, 11672–11679. https://doi.org/10.1021/acs.est.6b03260

Gionfriddo, C.M., Capo, E., Peterson, B., Heyu, L., Jones, D., Bravo, A.G., Bertilsson, S., Moreau, J., McMahon, K., Elias, D., Gilmour, C.C., 2021. Hg-MATE-Db.v1.01142021. Hg-cycling Microorg. Aquat. Terr. Ecosyst. database. https://doi.org/doi:10.25573/serc.13105370

Heimbürger, L.-E., Sonke, J. E., Cossa, D., Point, D., Lagane, C., Laffont, L., et al. (2015). Shallow methylmercury production in the marginal sea ice zone of the central Arctic Ocean. Scientific Reports, 5(1), 10318. https://doi.org/10.1038/srep10318

Hudson, J., Egan, S., 2022. Opportunistic diseases in marine eukaryotes: could Bacteroidota be the next threat to ocean life? Environ. Microbiol. 0–3. https://doi.org/10.1111/1462-2920.16094

Lee, S.H., Park, J.H., Kim, S.H., Yu, B.J., Yoon, J.J., Park, H.D., 2015. Evidence of syntrophic acetate oxidation by Spirochaetes during anaerobic methane production. Bioresour. Technol. 190, 543–549. https://doi.org/10.1016/j.biortech.2015.02.066

Li, H., 2013. Aligning sequence reads, clone sequences and assembly contigs with BWA-MEM. arXiv Prepr. arXiv:1303, 1–3.

Magoč, T., Salzberg, S.L., 2011. FLASH: Fast length adjustment of short reads to improve genome assemblies. Bioinformatics 27, 2957–2963. https://doi.org/10.1093/bioinformatics/btr507

Mahé, F., Czech, L., Stamatakis, A., Quince, C., de Vargas, C., Dunthorn, M., Rognes, T., 2021. Swarm v3: towards tera-scale amplicon clustering. Bioinformatics 38, 267–269. https://doi.org/10.1093/bioinformatics/btab493

Martin, M., 2011. Cutadapt removes adapter sequences from high-throughput sequencing reads. EMBnet J. 17, 10–12.

Matsen, F.A., Kodner, R.B., Armbrust, E.V., 2010. pplacer: linear time maximum-likelihood and Bayesian phylogenetic placement of sequences onto a fixed reference tree. BMC Bioinformatics 11, 538. https://doi.org/10.1186/1471-2105-11-538

Nurk, S., Meleshko, D., Korobeynikov, A., Pevzner, P.A., 2017. MetaSPAdes: A new versatile metagenomic assembler. Genome Res. 27, 824–834. https://doi.org/10.1101/gr.213959.116

Tada, Y., Marumoto, K., & Takeuchi, A. (2020). Nitrospina-like bacteria are potential mercury methylators in the mesopelagic zone in the East China Sea. *Frontiers in Microbiology*, *11*, 1369.

Van Vliet, D.M., Ayudthaya, S.P.N., Diop, S., Villanueva, L., Stams, A.J.M., Sánchez-Andrea, I., 2019. Anaerobic degradation of sulfated polysaccharides by two novel Kiritimatiellales strains isolated from black sea sediment. Front. Microbiol. 10, 1–16. https://doi.org/10.3389/fmicb.2019.00253

van Vliet, D.M., Lin, Y., Bale, N.J., Koenen, M., Villanueva, L., Stams, A.J.M., Sánchez-Andrea, I., 2020. Pontiella desulfatans gen. Nov., sp. nov., and pontiella sulfatireligans sp. nov., two marine anaerobes of the pontiellaceae fam. nov. producing sulfated glycosaminoglycan-like exopolymers. Microorganisms 8, 1–22. https://doi.org/10.3390/microorganisms8060920

Veldkamp, H., 1960. Isolation and characteristics of Treponema zuelzerae nov. spec., an anaerobic, free-living spirochete. Antonie Van Leeuwenhoek 26, 103–125. https://doi.org/10.1007/BF02538999

Villar, E., Cabrol, L., & Heimbürger‐Boavida, L. E. (2020). Widespread microbial mercury methylation genes in the global ocean. *Environmental Microbiology Reports*, *12*(3), 277-287.

Waite, D.W., Chuvochina, M., Pelikan, C., Parks, D.H., Yilmaz, P., Wagner, M., Loy, A., Naganuma, T., Nakai, R., Whitman, W.B., Hahn, M.W., Kuever, J., Hugenholtz, P., 2020. Proposal to reclassify the proteobacterial classes deltaproteobacteria and oligoflexia, and the phylum thermodesulfobacteria into four phyla reflecting major functional capabilities. Int. J. Syst. Evol. Microbiol. 70, 5972–6016. https://doi.org/10.1099/ijsem.0.004213

Wiegand, S., Jogler, M., Jogler, C., 2018. On the maverick Planctomycetes. FEMS Microbiol. Rev. 42, 739–760. https://doi.org/10.1093/femsre/fuy029

Yamada, T., Sekiguchi, Y., Hanada, S., Imachi, H., Ohashi, A., Harada, H., Kamagata, Y., 2006. Anaerolinea thermolimosa sp. nov., Levilinea saccharolytica gen. nov., sp. nov. and Leptolinea tardivitalis gen. nov., sp. nov., novel filamentous anaerobes, and description of the new classes Anaerolineae classis nov. and Caldilineae classis nov. in the . Int. J. Syst. Evol. Microbiol. 56, 1331–1340. https://doi.org/10.1099/ijs.0.64169-0
